# Transethnic insight into the genetics of glycaemic traits: fine-mapping results from the Population Architecture using Genomics and Epidemiology (PAGE) consortium

**DOI:** 10.1007/s00125-017-4405-1

**Published:** 2017-09-13

**Authors:** Stephanie A. Bien, James S. Pankow, Jeffrey Haessler, Yinchang N. Lu, Nathan Pankratz, Rebecca R. Rohde, Alfred Tamuno, Christopher S. Carlson, Fredrick R. Schumacher, Petra Bůžková, Martha L. Daviglus, Unhee Lim, Myriam Fornage, Lindsay Fernandez-Rhodes, Larissa Avilés-Santa, Steven Buyske, Myron D. Gross, Mariaelisa Graff, Carmen R. Isasi, Lewis H. Kuller, JoAnn E. Manson, Tara C. Matise, Ross L. Prentice, Lynne R. Wilkens, Sachiko Yoneyama, Ruth J. F. Loos, Lucia A. Hindorff, Loic Le Marchand, Kari E. North, Christopher A. Haiman, Ulrike Peters, Charles Kooperberg

**Affiliations:** 1 Division of Public Health Sciences, Fred Hutchinson Cancer Research Center, 1100 Fairview Ave N., Seattle, WA 98109-1024 USA; 2 Division of Epidemiology and Community Health, University of Minnesota, Minneapolis, MN USA; 3 Department of Biological Sciences, Vanderbilt University, Nashville, TN USA; 4 Department of Laboratory Medicine and Pathology, University of Minnesota, Minneapolis, MN USA; 5 Department of Epidemiology, School of Public Health, University of North Carolina at Chapel Hill, Chapel Hill, NC USA; 6 The Department of Preventive Medicine, The Icahn School of Medicine at Mount Sinai, New York, NY USA; 7 Department of Epidemiology and Biostatistics, Case Western Reserve University, Cleveland, OH USA; 8 Department of Biostatistics, University of Washington, Seattle, WA USA; 9 Department of Medicine, Institute for Minority Health Research, University of Illinois at Chicago, Chicago, IL USA; 10 Epidemiology Program, University of Hawaii Cancer Center, Honolulu, HI USA; 11 Human Genetics Center, University of Texas Health Science Center at Houston, Houston, TX USA; 12 Division of Cardiovascular Sciences, National Heart, Lung, and Blood Institute, National Institutes of Health, Bethesda, MD USA; 13 Department of Genetics, Rutgers University, Piscataway, NJ USA; 14 Department of Statistics, Rutgers University, Newark, NJ USA; 15 Department of Epidemiology & Population Health, Albert Einstein College of Medicine, Bronx, NY USA; 16 Department of Epidemiology, University of Pittsburgh, Pittsburgh, PA USA; 17 Department of Epidemiology, Harvard T.H. Chan School of Public Health, Boston, MA USA; 18 Department of Ophthalmology and Visual Sciences, University of Michigan, Ann Arbor, MI USA; 19 Department of Epidemiology, University of Michigan, Ann Arbor, MI USA; 20 MRC Epidemiology Unit, Institute of Metabolic Science, University of Cambridge, Cambridge, UK; 21 The Charles Bronfman Institute for Personalized Medicine, The Icahn School of Medicine at Mount Sinai, New York, NY USA; 22 The Icahn School of Medicine at Mount Sinai, New York, NY USA; 23 National Human Genome Research Institute, National Institutes of Health, Bethesda, MD USA; 24 Carolina Center for Genome Sciences, University of North Carolina at Chapel Hill, Chapel Hill, NC USA; 25 Department of Preventive Medicine, Keck School of Medicine, University of Southern California/Norris Comprehensive Cancer Center, Los Angeles, CA USA

**Keywords:** Fine-mapping, Genetic, Glucose, Glycaemic, Insulin, Multiethnic, Page, Transethnic, Type 2 diabetes

## Abstract

**Aims/hypothesis:**

Elevated levels of fasting glucose and fasting insulin in non-diabetic individuals are markers of dysregulation of glucose metabolism and are strong risk factors for type 2 diabetes. Genome-wide association studies have discovered over 50 SNPs associated with these traits. Most of these loci were discovered in European populations and have not been tested in a well-powered multi-ethnic study. We hypothesised that a large, ancestrally diverse, fine-mapping genetic study of glycaemic traits would identify novel and population-specific associations that were previously undetectable by European-centric studies.

**Methods:**

A multiethnic study of up to 26,760 unrelated individuals without diabetes, of predominantly Hispanic/Latino and African ancestries, were genotyped using the Metabochip. Transethnic meta-analysis of racial/ethnic-specific linear regression analyses were performed for fasting glucose and fasting insulin. We attempted to replicate 39 fasting glucose and 17 fasting insulin loci. Genetic fine-mapping was performed through sequential conditional analyses in 15 regions that included both the initially reported SNP association(s) and denser coverage of SNP markers. In addition, Metabochip-wide analyses were performed to discover novel fasting glucose and fasting insulin loci. The most significant SNP associations were further examined using bioinformatic functional annotation.

**Results:**

Previously reported SNP associations were significantly replicated (*p* ≤ 0.05) in 31/39 fasting glucose loci and 14/17 fasting insulin loci. Eleven glycaemic trait loci were refined to a smaller list of potentially causal variants through transethnic meta-analysis. Stepwise conditional analysis identified two loci with independent secondary signals (*G6PC2*-rs477224 and *GCK-*rs2908290), which had not previously been reported. Population-specific conditional analyses identified an independent signal in *G6PC2* tagged by the rare variant rs77719485 in African ancestry. Further Metabochip-wide analysis uncovered one novel fasting insulin locus at *SLC17A2*-rs75862513.

**Conclusions/interpretation:**

These findings suggest that while glycaemic trait loci often have generalisable effects across the studied populations, transethnic genetic studies help to prioritise likely functional SNPs, identify novel associations that may be population-specific and in turn have the potential to influence screening efforts or therapeutic discoveries.

**Data availability:**

The summary statistics from each of the ancestry-specific and transethnic (combined ancestry) results can be found under the PAGE study on dbGaP here: https://www.ncbi.nlm.nih.gov/projects/gap/cgi-bin/study.cgi?study_id=phs000356.v1.p1

**Electronic supplementary material:**

The online version of this article (doi:10.1007/s00125-017-4405-1) contains peer-reviewed but unedited supplementary material, which is available to authorised users.

## Introduction

Type 2 diabetes is a growing epidemic that disproportionally burdens US minority populations [1]. Elevated levels of fasting glucose and fasting insulin in individuals without diabetes are markers of dysregulated glucose metabolism and are strong risk factors for type 2 diabetes [2]. Although twin and family studies provide heritability estimates of 10–50% for these traits [3, 4], family-based linkage studies have been largely unsuccessful in identifying specific contributing loci. Genome-wide association studies (GWAS) greatly accelerated the pace of discovery of genetic variants contributing to glycaemic traits. For example, the Meta-Analyses of Glucose and Insulin-related traits (MAGIC) consortium performed a large-scale investigation of glycaemic traits in individuals of European descent without diabetes and identified 24 fasting glucose loci and eight fasting insulin loci, three of which were associated with both traits [5, 6]. These findings have implicated genes and pathways known to be related to glucose metabolism (e.g. *GCK*/*G6PC2* and glucose dephosphorylation), as well as novel pathways (e.g. *MTNR1B* and circadian rhythmicity). However, in some instances, the interpretation of GWAS findings has been challenging. For instance, many of the known loci are positioned in non-coding, putative regulatory regions of the genome, which in turn makes it difficult to identify the gene target(s). Additionally, the most significant variant is often not the causal variant but is a correlated variant in linkage disequilibrium with the functional variant(s).

While early GWAS efforts were focused on populations of European descent, initial attempts to generalise GWAS findings to more diverse populations have had limited success [7–9]. Importantly, these studies tended to be small and only included the initial most significant GWAS variant (index SNP). However, it is critical that transethnic investigation of GWAS loci include both the index variant and all correlated variants, given that patterns of linkage disequilibrium vary by ancestry and the functional SNP(s) are rarely known. On average, European populations have more highly correlated SNPs and extended haplotypes in comparison with populations of African ancestry (AA). Hispanic/Latino (H/L) populations, on the other hand, are more admixed with highly variable contributions of African, European and New World ancestry. Due in part to reduction in linkage disequilibrium with neighbouring SNPs, transethnic studies can utilise these differences across and within admixed populations to localise causal variants, and discover novel population-specific associations that were undetectable in genetically homogeneous studies. Thus, transethnic studies may provide insight into the underlying biology of complex traits, which may differ among groups.

The Metabochip was developed to fine-map GWAS loci for metabolic and cardiovascular traits, as well as replicate promising loci with suggestive, but not genome-wide, significant *p* values [10]. Among the 196,725 Metabochip variants selected for fine-mapping metabolic and cardiovascular-related loci, approximately 40,000 were selected for type 2 diabetes and related biomarkers. Among the 39 fasting glucose loci and 17 fasting insulin loci [5, 6] that were available for replication, 15 loci included not only the index SNP but also denser coverage of SNPs on the Metabochip that could be utilised for fine-mapping. Importantly, despite very large sample sizes, attempted Metabochip fine-mapping in a population of European descent generally did not yield stronger associations than the original GWAS index SNP and did not reduce the number of SNPs reaching similar levels of significance [11]. As such, this effort was unable to narrow in on functional candidate SNP(s).

This study examined the association of Metabochip SNPs with fasting glucose and fasting insulin in a multiethnic study of up to 26,760 participants: 14,953 H/L, 10,380 AA, 998 Asian and Pacific Islander (ASN) and 429 American Indian/Alaskan Native (AI/AN) populations from the Population Architecture using Genetic Epidemiology (PAGE) consortium. Specifically, we carried out the following procedures: (1) tested the association of index SNPs previously reported for 39 fasting glucose and 17 fasting insulin loci from studies of individuals of European descent; (2) used transethnic meta-analysis to refine known glycaemic trait loci in 15 loci which were densely covered with SNPs on the Metabochip; (3) investigated remaining metabolic and cardiovascular trait loci on the Metabochip for association with these glycaemic traits and (4) performed bioinformatic functional annotation of the most significant (lead) SNPs to further prioritise likely causal variants.

## Methods

### Ethics statement

This study was performed in accordance with the tenets of the Declaration of Helsinki and approved by the Institutional Review Boards of each participating study. All study participants provided written informed consent.

### Study population and trait measurement

The PAGE consortium was funded by the National Human Genome Research Institute (NHGRI) to investigate the epidemiological architecture of well-replicated genetic variants associated with human diseases or traits [12]. This analysis includes self-reported H/L, AA, ASN and AI/AN individuals without diabetes, aged 18 years or over, from the Multiethnic Cohort Study (MEC), the Women’s Health Initiative (WHI), Atherosclerosis Risk in Communities (ARIC), Coronary Artery Risk Development in Young Adults (CARDIA), the Hispanic Community Health Study/Study of Latinos (HCHS/SOL) and the Mount Sinai School of Medicine’s (MSSM) DNA biobank (Bio*Me*). Further details about each cohort can be found in the electronic supplementary materials (ESM) Methods (study population and trait measurement section).

Fasting glucose and fasting insulin concentrations were measured using standard assays, at laboratories specific to each PAGE site (ESM Table 1). Individuals self-reporting that they had ever been diagnosed with diabetes or taken diabetes medications or who had fasting blood glucose levels ≥ 6.99 mmol/l (≥ 126 mg/dl) were excluded from analyses. Individuals with BMI < 16.5 kg/m^2^ or BMI > 70 kg/m^2^ were also excluded on the assumption that these extremes could be attributable to data coding errors or underlying illness or could reflect a familial syndrome. Prior to analyses, each study removed race/ethnicity outliers using ancestry informative principal components.

After exclusions, fasting glucose analyses consisted of 14,953 H/L, 10,380 AA, 998 ASN and 429 AI/AN individuals. Fasting insulin analyses involved fewer individuals: 12,895 H/L, 8361 AA, 998 ASN and 420 AI/AN. Fasting insulin was not available for Bio*Me*. Race/ethnicity was self-reported. Descriptive characteristics of PAGE study participants by cohort can be found in ESM Table 2. While ASN and AI/AN were included for transethnic meta-analysis, population-specific analyses were underpowered due to small sample sizes. As such, ASN and AI/AN population-specific analyses were used as a comparison for consistency in the direction of effect.

### Genotyping and quality control

Genotyping was performed using the Metabochip, the design of which has been described elsewhere [10]. In brief, the 200K Metabochip is designed to cost effectively analyse putative association signals identified through GWAS of many glucose- and insulin-related metabolic and cardiovascular traits and to fine-map established loci [10]. More than 122,000 SNPs were included to fine-map 257 GWAS loci for 23 traits [10]. Fine-mapping loci were defined as the GWAS index SNP and all correlated SNPs (*r* ^2^ ≥ 0.5) that were within 0.02 cM of the index and having a minor allele frequency (MAF) > 1% in at least one HapMap Phase I population. SNPs were excluded if the Illumina design score was < 0.5 or there were SNPs within 15 bp of the SNP of interest with MAF of > 2% among Europeans (CEU [HapMap Population Code for Utah residents (CEPH) with Northern and Western European ancestry]).

Metobochip genotyping was performed for MEC, ARIC, CARDIA, HCHS/SOL and WHI [13] individuals. Standard quality control filters were applied for samples and SNPs, including missing rate and Hardy–Weinberg equilibrium (*p* < 1 × 10^−7^). A portion of WHI individuals of AA had both Metabochip and the Affymetrix 6.0 genotype data available from the SNP Health Association Resource (SHARe); this was used to impute Metabochip SNPs in the remaining SHARe participants with only Affymetrix 6.0 GWAS [8] and only dosages with imputation *R* ^2^ > 0.3 were included in the analyses. In Bio*Me*, genotypes from the Illumina HumanOmniExpress array were imputed to 1000 Genome Phase I haplotype panels (March 2012) [14]. Metabochip SNPs with ‘proper info’ score ≥ 0.4 were included in the analysis. Principal components were determined within each study using the Eigensoft software [15]. We excluded SNPs with a minor allele count less than 5 within each study by racial/ethnic population. The sample success rate and concordance rate for duplicate pairs across all studies was ≥ 95% and ≥ 99%, respectively. Further genotyping and analytical characteristics of the participating studies are further summarised in ESM Methods (genotyping and quality control section) and ESM Table 1.

### Replication and fine-mapping approach

**Fig. 1:**
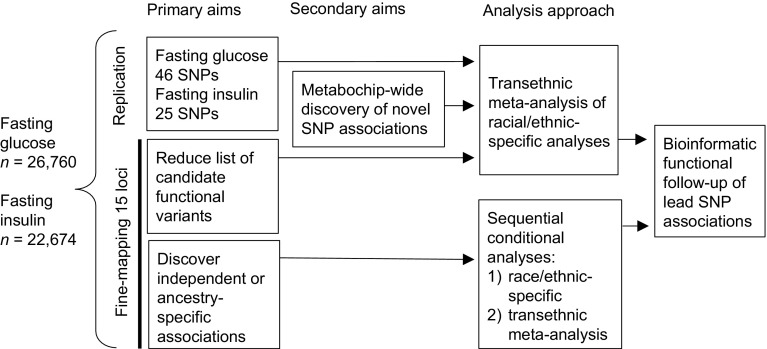
PAGE Metabochip Study Design. Primary results presented were from models including BMI as a covariate. ESM Tables 5 and 6 include results from models without BMI as a covariate

The overall study design for replication, fine-mapping and discovery of novel loci is summarised in Fig. 1. For replication of known loci, unconditional association analyses were performed for previously reported index SNPs listed in ESM Table 3. A nominal significance level (α = 0.05) was used to define replication of a locus. Next, unconditional association analyses were performed for all SNPs in a locus by race/ethnicity and by transethnic meta-analysis. A locus-specific *p* value threshold was defined as 0.05 divided by the number of SNPs passing quality control in each region (ranging from α = 1.4 × 10^−5^ to α = 4.1 × 10^−4^, Table 1). Locus-specific significance was used to conservatively adjust for multiple testing, while also acknowledging that genetic variation is known to influence glycaemic traits in these regions. Linkage disequilibrium was calculated for PAGE H/L, AA and Asian samples with 500 kb sliding windows using PLINK [16]. Metabochip linkage disequilibrium and frequency information in Europeans was provided by the 1000 Genomes Phase 3 population. These linkage disequilibrium patterns were used to evaluate locus refinement. Additionally, LocusZoom plots [17] were used to graphically display the fine-mapping results and linkage disequilibrium for these plots used 1000 Genomes Phase I Super Populations (European ancestry [EUR], admixed American ancestry [AMR], African ancestry [AFR]). After identifying the most significant lead SNP in each region, we searched for additional independent association signals by including the lead SNP in the conditional model and then testing each of the remaining SNPs in a region. These conditional analyses were repeated, adding in the lead SNP and conditional lead SNP(s), until no SNP in the model had a conditional *p* value less than the locus-specific significance. Sequential conditional analyses were performed for each race/ethnicity and transethnic meta-analysis. Further details on our approach to locus refinement are provided in ESM Methods (replication and fine-mapping of known glycaemic trait loci section).

**Table 1:** Characterisation of 15 fine-mapping genomic regions analysed for fasting glucose and fasting insulin

Chromosome	Locus	Base pair range (GRCh37/hg19)	No. of SNPs on Metabochip	No. of SNPs^a^	α	Trait
1q32.3	*PROX1*	214,124,818–214,167,508	153	129	3.9 × 10^−4^	Glucose
2p23.3	*GCKR*	27,389,634–27,951,658	1099	966	5.2 × 10^−5^	Both
2q31.1	*G6PC2*	169,752,640–169,814,655	240	211	2.4 × 10^−4^	Glucose
3q21.1	*ADCY5*	122,976,919–123,206,919	924	786	6.2 × 10^−5^	Glucose
3q26.2	*SLC2A2*	170,532,111–170,769,171	717	653	7.7 × 10^−5^	Glucose
7p21.2	*DGKB*	14,185,088–15,145,520	3894	3555	1.4 × 10^−5^	Glucose
7p13	*GCK*	44,222,003–44,266,077	148	122	4.1 × 10^−4^	Glucose
9p24.2	*GLIS3*	4,243,162–4,310,558	419	385	1.3 × 10^−4^	Glucose
10q25.2	*ADRA2A/TCF7L2*	112,967,738–113,053,039	462	424	1.2 × 10^−4^	Glucose
11p15.4	*CRY2*	45,706,162–46,162,829	1082	921	5.4 × 10^−5^	Glucose
11p11.2	*MADD*	46,921,641–48,091,303	2392	2037	2.5 × 10^−5^	Glucose
11q12.2	*FADS2*	61,505,583–61,751,624	726	643	7.8 × 10^−5^	Glucose
11q14.3	*MTNR1B*	92,667,047–92,725,321	214	180	2.8 × 10^−4^	Glucose
12q23.2	*IGF1*	103,851,897–104,450,976	1307	1059	4.7 × 10^−5^	Insulin
15q22.2	*C2CD4A*	62,099,182–62,520,109	1143	949	5.3 × 10^−5^	Glucose

α is the Bonferroni significance threshold (0.05/no. of SNPs passing quality control) used to define region-specific significance

^a^No. of SNPs passing quality control in the transethnic meta-analysis

### Discovery of novel loci

Metabochip-wide analyses were performed to identify novel associations with fasting glucose and fasting insulin. Statistical significance for the Metabochip-wide analysis was set at 0.05 divided by the number of Metabochip SNPs passing quality control (α = 2.7 × 10^−7^). Results were examined through qq plots and Manhattan plots for each model, highlighting known regions defined in ESM Table 4. Further details are provided in ESM Methods (strategy for selecting novel associations section).

### Statistical analysis

First, in each study with unrelated individuals we performed race/ethnic-specific analyses for fasting glucose and natural log-transformed fasting insulin, excluding ancestry outliers and first-degree relatives. In HCHS/SOL, a weighted version of generalised estimation equations was used to account for unequal inclusion probabilities and complex family-based sampling designs [18]. Models adjusted for age, sex (except WHI), study site (as applicable), smoking status (current vs former/never), continuous BMI and ancestry principal components. Like previous studies [11], primary analyses adjusted for BMI because it is a major risk factor for type 2 diabetes and is correlated with glycaemic traits. For comparison, all models were also run without adjustment for BMI. Next, fixed-effect models with inverse-variance weighting were used to pool the study-specific SNP effect estimates and their standard errors by race/ethnicity as implemented in METAL [19]. Finally, summary statistics from METAL for H/L, AA, NA/AI and ASN were combined using inverse-variance weighted fixed effects meta-analysis in METAL. *Q* statistics and *I* ^2^ were used to evaluate heterogeneity across studies and race/ethnicity. Further details are provided in ESM Methods (statistical analysis section).

### Functional annotation

Detailed information on the functional annotation methods and various datasets used is provided in ESM Methods (functional annotation section). In brief, it is expected that the lead SNPs are more likely to be functional or to be in stronger linkage disequilibrium with underlying functional variant(s). Therefore, lead SNPs and all correlated SNPs (*r* ^2^ > 0.2 in 1000 Genomes Phase 3 AFR/AMR populations) were annotated using publicly available functional datasets. Potential functional effects were assessed using PolyPhen2 [20] (http://genetics.bwh.harvard.edu/pph2/, accessed 24 August 2016) for non-synonymous variants, SPANR (http://tools.genes.toronto.edu/) [21] for variants near splice sites, TargetScan miRNA Regulatory Sites for 3′-UTR regions [22], ENCODE/NIH Roadmap data [23–25] and GTEx (https://www.gtexportal.org/home/) [26] to identify non-coding variants positioned in predicted regulatory elements.

## Results

### Demographics

We included a total of 26,760 participants (14,953 H/L, 10,380 AA, 998 ASN, and 429 AI/AN) in fasting glucose analyses. The sample sizes for fasting insulin analyses were slightly smaller, with a total of 22,674 participants (12,895 H/L, 8361 AA, 998 ASN and 420 AI/AN). The mean age across the five cohorts was 55 years for men and 59 years for women (range 18–93 years). Study-specific descriptive characteristics are shown in ESM Table 2. Particularly due to the inclusion of the WHI cohort, the proportion of women in the total study population was high, with the highest fraction observed in AA (82.6% for fasting glucose and 97.1% for fasting insulin). Glycaemic trait distributions were similar across studies and ethnicities, with average fasting glucose levels ranging from 4.7 ± 0.7 mmol/l to 5.5 ± 0.6 mmol/l and average fasting insulin levels ranging from 43.3 ± 23.6 pmol/l to 75.9 ± 38.8 pmol/l.

### Generalisation of European glycaemic trait loci

We found that 31/39 (79.5%) fasting glucose loci and 14/17 (82.3%) fasting insulin loci had a *p* value smaller than 0.05. Index SNP associations were directionally consistent in our transethnic PAGE meta-analysis and only four SNPs had heterogeneity *p* values less than 0.05 (Table 2). The effect estimates (βs) of index SNPs in the transethnic meta-analysis were very similar to those published in Metabochip analysis of individuals of European descent (Pearson’s *r* ^2^ = 0.86, 95% CI 0.78, 0.91; *p* < 2.2 × 10^−16^; ESM Fig. 1). At three loci (*WARS*, *GIPR* and *DPYSLS*) we observed replication in only H/L and not the transethnic meta-analysis. Interestingly, while the sample sizes were much smaller for Asian individuals than for H/L and AA individuals, the transethnic meta-analysis of the *PROX1* index (rs340874) was only nominally significant and directionally consistent in the Asian samples. In the remaining loci that did not replicate in transethnic meta-analysis or the race/ethnic-specific analyses, the effects were generally similar or at least in the same direction. Analyses without inclusion of BMI as a covariate were generally similar, with slightly lower significance at some loci. Full summary statistics for models with and without BMI covariate are reported in ESM Table 5 and ESM Table 6, respectively.

**Table 2:** Replication of European Metabochip index SNPs for 39 fasting glucose and 17 fasting insulin loci via transethnic meta-analysis

Locus/gene	Lead EUR	C/NC allele	Coded allele frequency					Effect β of coded allele (SE)					Analyses with *p* < 0.05	*p* value TE Meta (Het.)
			EUR	H/L	AA	ASN	TE Meta	EUR	H/L	AA	ASN	TE Meta		
Fasting glucose loci (N_TE_ = 26,760, N_EUR_ = 118,881)														
1q32.3 *PROX1*	rs340874	A/G	0.48	0.60	0.82	0.61	0.67	−0.015 (0.002)	−0.004 (0.006)	−0.009 (0.009)	0.076 (0.027)	−0.003 (0.005)	ASN	0.59 (0.02)
2p23.3 *GCKR*	rs780094	A/G	0.39	0.35	0.19	0.52	0.30	−0.029 (0.002)	−0.033 (0.007)	−0.016 (0.010)	−0.051 (0.027)	−0.029 (0.005)	H/L, ASN, TE	2 × 10^−8^ (0.2)
2q31.1 *G6PC2*	rs560887	A/G	0.30	0.17	0.07	0.03	0.14	−0.075 (0.003)	−0.086 (0.008)	−0.063 (0.014)	−0.065 (0.077)	−0.079 (0.007)	H/L, AA, TE	1 × 10^−29^ (0.48)
3q21.1 *ADCY5*	rs11708067	A/G	0.79	0.75	0.84	0.96	0.78	0.024 (0.003)	0.021 (0.007)	0.052 (0.010)	−0.254 (0.171)	0.031 (0.006)	H/L, AA, TE	5 × 10^−8^ (0.02)
3q26.2 *SLC2A2*	rs1280	A/G	0.86	0.84	0.65	0.97	0.73	0.031 (0.003)	0.052 (0.009)	−0.001 (0.007)	0.043 (0.082)	0.021 (0.006)	H/L, TE	1 × 10^−4^ (2 × 10^−5^)
7p21.2 *DGKB*	rs2191349	A/C	0.53	0.48	0.57	0.69	0.51	0.032 (0.002)	0.023 (0.006)	0.005 (0.009)	0.003 (0.028)	0.017 (0.005)	H/L, TE	8 × 10^−4^ (0.42)
7p13 *GCK*	rs730497	A/G	0.16	0.20	0.18	0.18	0.20	0.061 (0.003)	0.061 (0.008)	0.056 (0.009)	0.004 (0.034)	0.057 (0.006)	H/L, AA, TE	3 × 10^−22^ (0.37)
8q24.11 *SLC30A8*	rs11558471	A/G	0.68	0.75	0.90	0.57	0.77	0.032 (0.002)	0.018 (0.007)	0.014 (0.012)	−0.004 (0.026)	0.017 (0.006)	H/L, TE	4 × 10^−3^ (0.22)
9p24.2 *GLIS3*	rs10814916	A/C	0.49	0.43	0.33	0.54	0.40	−0.017 (0.002)	−0.016 (0.006)	−0.009 (0.008)	−0.066 (0.027)	−0.015 (0.005)	H/L, ASN, TE	1 × 10^−3^ (0.21)
10q25.2 *ADRA2A*	rs11195502	A/G	0.09	0.13	0.34	0.07	0.25	−0.036 (0.004)	−0.014 (0.010)	−0.012 (0.008)	−0.022 (0.054)	−0.013 (0.006)	TE	0.04 (0.62)
10q25.2 *TCF7L2*	rs4506565	A/T	0.70	0.71	0.56	0.93	0.64	−0.024 (0.002)	−0.030 (0.007)	−0.019 (0.007)	−0.137 (0.060)	−0.025 (0.005)	All	3 × 10^−7^ (0.19)
11p11.2 *CRY2*	rs11605924	A/C	0.49	0.54	0.86	0.81	0.63	0.022 (0.002)	0.017 (0.006)	0.027 (0.011)	−0.066 (0.034)	0.018 (0.005)	All	1 × 10^−3^ (0.03)
11p11.2 *MADD*	rs11039182	A/G	0.73	0.82	0.95	0.97	0.85	0.023 (0.003)	0.000 (0.009)	0.021 (0.016)	−0.002 (0.091)	0.004 (0.007)	None	0.55 (0.67)
11q12.2 *FADS2*	rs174550	A/G	0.66	0.52	0.91	0.57	0.60	0.018 (0.002)	0.026 (0.007)	0.036 (0.013)	0.039 (0.027)	0.029 (0.006)	H/L, AA, TE	7 × 10^−7^ (0.9)
11q14.3 *MTNR1B*	rs10830963	C/G	0.71	0.79	0.93	0.60	0.81	−0.078 (0.003)	−0.062 (0.008)	−0.090 (0.014)	−0.078 (0.026)	−0.068 (0.006)	All	7 × 10^−27^ (0.21)
15q22.2 *C2CD4A*	rs4502156	A/G	0.55	0.40	0.26	0.52	0.35	0.023 (0.002)	0.017 (0.007)	0.006 (0.008)	0.008 (0.026)	0.012 (0.005)	H/L, TE	0.01 (0.77)
9p21.3 *CDKN2B*	rs10811661	A/G	0.82	0.86	0.93	0.56	0.86	0.024 (0.003)	0.021 (0.009)	0.017 (0.014)	0.072 (0.026)	0.024 (0.007)	H/L, ASN, TE	0.02 (0.29)
5q15 *PCSK1*	rs4869272	A/G	0.69	0.75	0.78	0.73	0.76	0.018 (0.002)	0.021 (0.007)	0.019 (0.008)	0.032 (0.029)	0.020 (0.005)	H/L, AA, TE	1 × 10^−3^ (0.97)
13q12.2 *PDX1*	rs11619319	A/G	0.77	0.71	0.83	0.55	0.75	−0.020 (0.002)	−0.008 (0.007)	−0.017 (0.010)	−0.054 (0.026)	−0.012 (0.006)	AA, ASN, TE	0.05 (0.32)
8p23.1 *PPP1R3B*	rs983309	A/C	0.12	0.21	0.28	0.02	0.24	0.026 (0.003)	0.023 (0.008)	0.017 (0.008)	0.004 (0.104)	0.020 (0.006)	H/L, AA, TE	2 × 10^−3^ (0.96)
7p12.1 *GRB10*	rs6943153	A/G	0.34	0.45	0.68	0.28	0.54	0.015 (0.002)	0.019 (0.006)	−0.004 (0.008)	−0.010 (0.030)	0.009 (0.005)	H/L, TE	0.07 (0.11)
11q13.4 *ARAP1*	rs11603334	A/G	0.17	0.08	0.05	0.05	0.07	−0.019 (0.003)	−0.030 (0.011)	−0.039 (0.016)	−0.086 (0.067)	−0.033 (0.009)	H/L, AA, TE	1 × 10^−5^ (0.69)
20p11.21 *FOXA2*	rs6113722	A/G	0.04	0.05	0.16	0.18	0.13	−0.035 (0.005)	−0.042 (0.014)	−0.040 (0.010)	−0.090 (0.033)	−0.043 (0.008)	All	2 × 10^−6^ (0.55)
9q31.3 *IKBKAP*	rs16913693	A/C	0.97	0.96	0.77	1	0.81	0.043 (0.007)	0.010 (0.017)	−0.012 (0.008)	0.334 (0.333)	−0.008 (0.008)	None	0.51 (0.48)
9q34.3 *DNLZ*	rs3829109	A/G	0.29	0.33	0.17	0.13	0.28	−0.017 (0.003)	−0.021 (0.007)	−0.026 (0.010)	0.000 (0.040)	−0.022 (0.006)	H/L, AA, TE	5 × 10^−4^ (0.91)
14q32.2 *WARS*	rs3783347	A/C	0.21	0.12	0.06	0.1	0.11	−0.017 (0.003)	−0.023 (0.010)	0.000 (0.014)	0.000 (0.044)	−0.014 (0.008)	H/L	0.08 (0.40)
19q13.32 *GIPR*	rs2302593	C/G	0.5	0.51	0.28	0.39	0.42	0.014 (0.002)	−0.013 (0.006)	−0.002 (0.008)	0.019 (0.027)	−0.008 (0.005)	H/L	0.05 (0.55)
6p22.3 *CDKAL1*	rs9368222	A/C	0.28	0.23	0.19	0.41	0.23	0.014 (0.002)	0.025 (0.007)	0.025 (0.009)	0.041 (0.026)	0.026 (0.006)	H/L, AA, TE	3 × 10^−5^ (0.94)
12q24.33 *P2RX2*	rs10747083	A/G	0.66	0.69	0.85	0.83	0.74	0.013 (0.002)	0.010 (0.007)	0.012 (0.011)	−0.017 (0.034)	0.010 (0.006)	None	0.12 (0.88)
20q12 *TOP1*	rs6072275	A/G	0.16	0.12	0.08	0.02	0.11	0.016 (0.003)	0.021 (0.010)	0.019 (0.013)	−0.075 (0.121)	0.021 (0.008)	H/L, TE	5 × 10^−3^ (0.53)
3q27.2 *IGF2BP2*	rs7651090	A/G	0.69	0.7	0.46	0.7	0.59	−0.013 (0.002)	−0.011 (0.007)	−0.011 (0.007)	−0.023 (0.029)	−0.011 (0.005)	TE	0.07 (0.90)
13q13.1 *KL*	rs576674	A/G	0.85	0.68	0.4	0.85	0.56	−0.017 (0.003)	−0.026 (0.007)	−0.014 (0.007)	0.054 (0.038)	−0.019 (0.005)	H/L, AA, TE	7 × 10^−4^ (0.08)
3p21.31 *AMT*	rs11715915	A/G	0.32	0.21	0.24	0.08	0.22	−0.012 (0.002)	−0.007 (0.008)	0.003 (0.008)	0.053 (0.051)	−0.002 (0.006)	None	0.59 (0.56)
6p24.3 *RREB1*	rs17762454	A/G	0.26	0.33	0.16	0.41	0.28	0.012 (0.002)	0.017 (0.007)	0.012 (0.010)	0.011 (0.027)	0.015 (0.005)	H/L, TE	0.02 (0.97)
5q13.3 *ZBED3*	rs7708285	A/G	0.73	0.69	0.85	0.91	0.74	−0.011 (0.003)	−0.004 (0.007)	0.003 (0.010)	0.002 (0.060)	−0.003 (0.006)	None	0.4 (0.47)
12q13.3 *GLS2*	rs2657879	A/G	0.82	0.81	0.93	NA	0.83	−0.012 (0.003)	−0.011 (0.008)	0.016 (0.015)	…	−0.005 (0.007)	None	0.43 (0.11)
2p23.3 *DPYSL5*	rs1371614	A/G	0.25	0.38	0.35	0.16	0.36	0.020 (0.004)	0.021 (0.007)	−0.006 (0.007)	−0.021 (0.036)	0.009 (0.005)	H/L	0.03 (0.05)
15q22.2 *C2CD4B*	rs12440695*	A/G	0.63	0.57	0.83	0.71	0.65	0.008 (0.003)	0.004 (0.007)	−0.002 (0.009)	−0.011 (0.028)	0.003 (0.005)	None	0.63 (0.58)
11p11.2 *OR4S1*	rs1483121	A/G	0.14	0.09	0.03	0.03	0.08	−0.027 (0.005)	0.008 (0.011)	−0.022 (0.022)	−0.101 (0.220)	0.002 (0.010)	None	0.59 (0.62)
Fasting insulin loci (N_TE_ = 22,674, N_EUR_ = 99,029)														
1q41 *LYPLAL1*	rs4846565	A/G	0.33	0.41	0.09	0.34	0.32	−0.013 (0.002)	−0.023 (0.008)	−0.007 (0.013)	0.022 (0.028)	−0.017 (0.007)	H/L, TE	0.01 (0.34)
2p23.3 *GCKR*	rs780094	A/G	0.39	0.35	0.19	0.52	0.30	−0.029 (0.002)	−0.031 (0.008)	−0.029 (0.010)	−0.011 (0.027)	−0.030 (0.006)	H/L, AA, TE	2 × 10^−7^ (0.41)
2q24.3 *GRB14*	rs10195252	A/G	0.60	0.67	0.28	0.89	0.49	0.017 (0.002)	0.041 (0.008)	0.036 (0.008)	−0.044 (0.044)	0.037 (0.006)	H/L, AA, TE	1 × 10^−10^ (0.29)
2q36.3 *IRS1*	rs2943645	A/G	0.63	0.74	0.63	0.90	0.68	0.019 (0.002)	0.018 (0.009)	0.012 (0.008)	0.062 (0.046)	0.016 (0.006)	H/L, TE	4 × 10^−3^ (0.54)
3p25.2 *PPARG*	rs17036328	A/G	0.86	0.89	0.83	0.95	0.85	0.021 (0.003)	0.038 (0.012)	0.009 (0.010)	0.036 (0.068)	0.022 (0.007)	H/L, TE	2 × 10^−3^ (0.15)
4q22.1 *FAM13A*	rs3822072	A/G	0.48	0.44	0.51	0.63	0.47	0.012 (0.002)	0.008 (0.008)	0.018 (0.010)	0.024 (0.028)	0.012 (0.006)	AA, TE	0.04 (0.82)
4q24 *TET2*	rs974801	A/G	0.62	0.58	0.72	0.40	0.64	−0.014 (0.002)	−0.018 (0.008)	−0.009 (0.008)	−0.023 (0.027)	−0.015 (0.006)	H/L, TE	6 × 10^−3^ (0.31)
4q32.1 *PDGFC*	rs6822892	A/G	0.68	0.59	0.27	0.70	0.45	0.014 (0.002)	0.012 (0.008)	0.003 (0.008)	0.009 (0.029)	0.009 (0.006)	None	0.12 (0.76)
5q11.2 *ARL15*	rs4865796	A/G	0.67	0.79	0.75	0.81	0.77	0.015 (0.002)	0.016 (0.009)	0.024 (0.008)	0.006 (0.036)	0.020 (0.006)	AA, TE	9 × 10^−4^ (0.80)
5q11.2 *ANKRD55*	rs459193	A/G	0.27	0.27	0.42	0.52	0.36	−0.015 (0.002)	−0.025 (0.009)	−0.022 (0.008)	−0.040 (0.026)	−0.022 (0.006)	All	4 × 10^−5^ (0.30)
6p21.31 *UHRF1BP1*	rs6912327	A/G	0.80	0.69	0.35	NA	0.51	0.016 (0.003)	0.004 (0.008)	−0.004 (0.008)	…	0.001 (0.006)	None	0.83 (0.08)
6q22.33 *RSPO3*	rs2745353	A/G	0.51	0.58	0.60	0.61	0.59	0.011 (0.002)	0.016 (0.008)	0.010 (0.008)	−0.039 (0.027)	0.011 (0.005)	H/L, TE	0.03 (0.25)
7q11.23 *HIP1*	rs1167800	A/G	0.54	0.67	0.84	0.69	0.73	0.011 (0.002)	0.018 (0.008)	0.009 (0.010)	−0.004 (0.028)	0.011 (0.006)	H/L	0.08 (0.07)
8p23.1 *PPP1R3B*	rs983309	A/C	0.13	0.21	0.28	0.02	0.25	0.022 (0.003)	0.026 (0.010)	0.024 (0.008)	−0.082 (0.103)	0.026 (0.006)	All	2 × 10^−5^ (0.02)
10q25.2 *TCF7L2*	rs7903146	A/G	0.27	0.25	0.28	0.08	0.27	−0.013 (0.002)	−0.014 (0.009)	−0.022 (0.008)	0.023 (0.057)	−0.019 (0.006)	AA, TE	1 × 10^−3^ (0.51)
12q23.2 *IGF1*	rs35767	A/G	0.18	0.24	0.44	0.33	0.36	−0.003 (0.003)	−0.014 (0.011)	0.006 (0.008)	−0.050 (0.032)	−0.004 (0.006)	None	0.43 (0.28)
19q13.11 *PEPD*	rs731839	A/G	0.66	0.61	0.63	0.48	0.61	−0.015 (0.002)	−0.016 (0.008)	−0.003 (0.008)	−0.037 (0.026)	−0.012 (0.005)	H/L, TE	0.03 (0.23)

EUR, individuals of European descent from Scott et al. [11] genotyped on Metabochip. Models included continuous BMI covariate, *rs12440695 used as a linkage disequilibrium proxy (*r* ^2^ = 0.98) for the index SNP rs11071657, which did not pass quality control. β, allelic effect size for an additive genetic model corresponding to the coded (C) allele, is shown in units of mmol/l for fasting glucose and natural log-transformed pmol/l for fasting insulin. Full results for models with and without BMI covariate for fasting glucose and fasting insulin are shown in ESM Table 5 and ESM Table 6, respectively

*p* values are shown for the transethnic (TE) meta-analysis and heterogeneity (Het.) in effect across populations

### Fine-mapping of European glycaemic trait loci

Among the 15 glycaemic trait loci for which fine-mapping was attempted on the Metabochip, ten fasting glucose loci and two fasting insulin loci had one or more SNPs that reached locus-specific significance (α = 0.05/number of SNPs in the locus) in the transethnic meta-analysis. The *p* values ranged from 1.0 × 10^−29^ at *G6PC2*-rs560887 to 1.5 × 10^−4^ at *PROX1*-rs10494973 (Table 3). Although AI/AN ancestries were included in the transethnic meta-analysis, the AI/AN results are not shown because the small sample size was underpowered for population-specific analysis. At four fasting glucose loci, the most significant lead SNP in PAGE transethnic meta-analysis was the same as the European index SNP from prior Metabochip evaluation (*G6PC2*, *ADCY5*, *MTNR1B* and *FADS2*). For six fasting glucose loci (*PROX1*, *GCKR*, *SLC2A2*, *DGKB*, *GCK* and *GLIS3*) and the one fasting insulin locus (*GCKR*), the lead SNP in PAGE transethnic meta-analysis was in moderate or weak linkage disequilibrium with the index SNP in 1000 Genomes Population EUR (*r* ^2^ > 0.2). At these six fasting glucose loci and one fasting insulin locus, the PAGE lead SNP and EUR index SNP were not independent of each other as only one of the two SNP associations maintained nominal significance in transethnic conditional meta-analysis where both lead and index variants were included in the model. This was further supported by investigation of potential fine-mapping through locus zoom plots.

**Table 3:** Most significant lead SNPs in ten fasting glucose and two fasting insulin fine-mapping loci identified in transethnic meta-analysis

Region	Lead PAGE SNP	Frequency of coded (C) allele						Effect β of coded allele (SE)				*p* value		*r* ^2^ with EUR index SNP^c^					No. of LD SNPs^e^	
		C/N	TE^a^	EUR	H/L	AA	ASN	TE Meta	H/L	AA	ASN	TE Meta^b^	Het.	EUR SNP^d^	EUR	H/L	AA	ASN	EUR	TE (% red.)^f^
Fasting glucose loci																				
1q32.3 *PROX1*	rs10494973	C/G	0.03	0.48	0.03	0.01	0.01	0.060 (0.016)	0.050 (0.018)**	0.100 (0.036)**	−0.274 (0.384)	2 × 10^−4^	0.44	rs340874	<0.10	<0.10	<0.10	<0.10	4	1 (75)
2p23.3 *GCKR* ^†^	rs1260326	A/G	0.29	0.41	0.34	0.15	0.52	−0.032 (0.005)	−0.036 (0.007)***	−0.020 (0.010)*	−0.051 (0.026)*	2 × 10^−9^	0.44	rs780094	0.92	0.91	0.42	0.93	274	90 (67)
2q31.1 *G6PC2* ^†^	rs560887	A/G	0.14	0.31	0.17	0.07	0.03	−0.079 (0.007)	−0.086 (0.008)***	−0.063 (0.014)***	−0.065 (0.077)	1 × 10^−29^	0.48	Same	1	1	1	1	118	9 (92)
3q21.1 *ADCY5* ^†^	rs11708067	A/G	0.78	0.82	0.75	0.84	0.97	0.031 (0.006)	0.021 (0.007)**	0.052 (0.010)***	−0.254 (0.171)	5 × 10^−8^	0.02	Same	1	1	1	1	72	18 (75)
3q26.2 *SLC2A2* ^†^	rs1604038	A/G	0.44	0.29	0.34	0.58	0.23	−0.026 (0.005)	−0.031 (0.007)***	−0.023 (0.007)**	0.037 (0.032)	1 × 10^−7^	0.2	rs1280	0.38	0.45	0.34	0.09	318	162 (49)
7p21.2 *DGKB* ^†^	rs62448618	A/T	0.34	0.50	0.38	0.27	0.50	0.022 (0.005)	0.030 (0.007)***	0.014 (0.008)	−0.001 (0.026)	1 × 10^−5^	0.33	rs2191349	0.81	0.61	0.03	0.39	133	12 (91)
7p13 *GCK* ^†^	rs2908286	A/G	0.19	0.18	0.2	0.18	0.20	0.060 (0.006)	0.064 (0.008)***	0.061 (0.009)***	0.002 (0.032)	9 × 10^−25^	0.27	rs730497	0.99	0.9	0.52	0.91	25	18 (28)
9p24.2 *GLIS3* ^†^	rs10974438	A/C	0.76	0.62	0.71	0.86	0.63	−0.023 (0.006)	−0.019 (0.007)**	−0.021 (0.010)*	−0.080 (0.028)**	6 × 10^−5^	0.16	rs10814916	0.53	0.27	0.08	0.69	54	7 (87)
11q12.2 *FADS2* ^†^	rs174547	A/G	0.60	0.66	0.52	0.91	0.55	0.029 (0.006)	0.026 (0.007)***	0.038 (0.013)**	0.039 (0.027)	4 × 10^−7^	0.86	Same	1	1	1	1	147	44 (70)
11q14.3 *MTNR1B* ^†^	rs10830963	C/G	0.81	0.78	0.79	0.93	0.59	−0.068 (0.006)	−0.062 (0.008)***	−0.090 (0.014)***	−0.078 (0.026)**	7 × 10^−27^	0.21	Same	1	1	1	1	94	1 (99)
Fasting insulin loci																				
2p23.3 *GCKR* ^†^	rs1260326	A/G	0.29	0.41	0.35	0.16	0.52	−0.035 (0.006)	−0.034 (0.008)***	−0.034 (0.010)***	−0.010 (0.027)	1 × 10^−8^	0.20	rs780094	0.92	0.91	0.42	0.93	274	90 (67)
12q23.2 *IGF1*	rs10860845	A/C	0.6	0.83	0.48	0.74	0.65	−0.023 (0.006)	−0.025 (0.008)***	−0.023 (0.008)**	0.002 (0.028)	3 × 10^−5^	0.76	rs860598	<0.10	<0.10	<0.10	<0.10	322	64 (80)

β: effect size from an additive multivariate model including BMI and corresponding to the coded (C) allele, is shown in units of mmol/l for fasting glucose and natural log-transformed pmol/l for fasting insulin

^a^MAF averaged across ethnicities H/L, AI/AN and ASN from the transethnic (TE) meta-analysis for coded allele

^b^ *p* value from the transethnic meta-analysis

^c^Linkage disequilibrium calculated from 1000 genomes Phase 3 super populations (EUR, AFR, AMR, and ASN)

^d^European SNP index defined as most significant SNP from the Scott et al. [11] Metabochip analysis

^e^No. of SNPs in linkage disequilibrium using *r* ^2^ > 0.2 calculated from 1000 genomes Phase 3 super populations with transethnic equal to the intersect of SNPs in EUR, AFR, AMR and ASN

^f^Percentage reduction in the number of SNPs

*p < 0.05, **p < 0.01 and ***p < 0.001 for race/ethnic-specific analyses

^†^Significant at region-specific Bonferroni-corrected transethnic meta-analysis *p* values (ranging from α = 1.41 × 10^−5^ to α = 4.1 × 10^−4^)

EUR, Europeans, LD, linkage disequilibrium, TE, transethnic

**Fig. 2:**
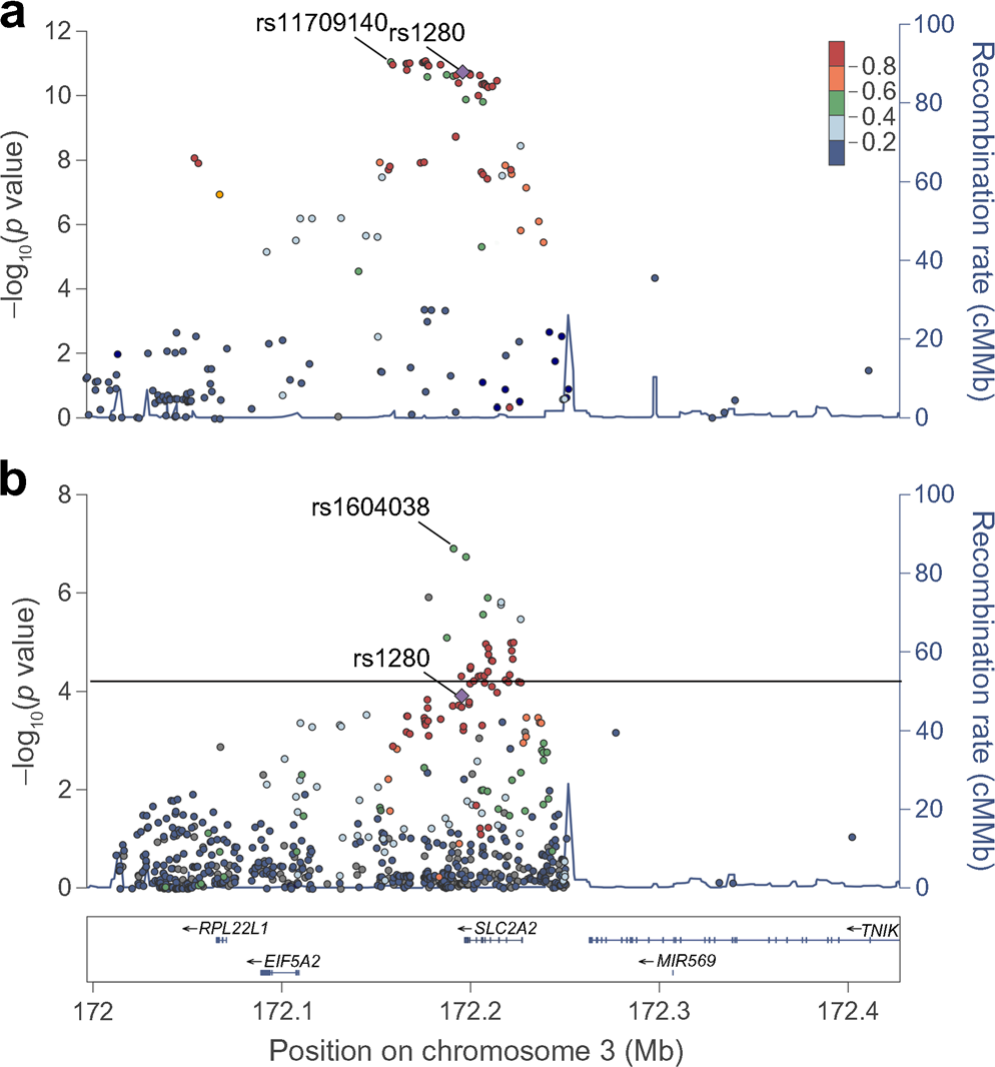
*SLC2A2* regional plot. Regional plots of SNP associations (−log_10_(*p* value)) with fasting glucose are shown for the MAGIC European (**a**) and the PAGE transethnic (**b**) meta-analyses. Not all SNPs used in the transethnic meta-analysis were present in the available MAGIC data (www.magicinvestigators.org/downloads/, accessed 26 June 2017) because of mapping issues [11]. SNPs not passing quality control or outside the fine-mapping region were removed from the transethnic plots. The colour scale indicates linkage disequilibrium (*r* ^2^) between each fine-mapping SNP and the GWAS index SNP (rs1280, purple diamond), which was calculated using 1000 Genomes Populations (CEU for MAGIC and AMR for PAGE). The population chosen for linkage disequilibrium colouring in the transethnic meta-analysis was based on population-specific analysis results (choosing the one with strongest underlying SNP associations). The most significant SNPs in MAGIC fine-mapping (rs11709140) and PAGE (rs1604038) are labelled

For each of the 11 glycaemic trait loci with potential transethnic fine-mapping (fasting glucose loci–*PROX1*, *G6PC2*, *ADCY5*, *MTNR1B*, *FADS2*, *GCKR*, *SLC2A2*, *DGKB*, *GCK* and *GLIS3*; fasting insulin locus–*GCKR*), we found that the number of SNPs in linkage disequilibrium with the most significant marker in the transethnic results (*r* ^2^ ≥ 0.2 in the 1KG super populations AFR and AMR) were less than the number of SNPs tagged by the EUR marker (*r* ^2^ ≥ 0.2 in EUR). Visual inspection of locus zoom plots indicated that transethnic meta-analysis refined each of these loci by reducing the number of highly correlated SNPs reaching the same level of significance and/or narrowing the genomic region containing putative causal SNPs (ESM Fig. 2). On average, the number of variants in high linkage disequilibrium was reduced by 72.5% with the number of linkage disequilibrium SNPs ranging from one at *MTNR1B* to 162 at *SLC2A2* in the PAGE transethnic meta-analysis results. Refinement was most evident at the *SLC2A2* locus (Fig. 2). Bioinformatic functional follow-up was performed for each of the eleven glycaemic trait loci with one or more variants passing the region-specific significance threshold in our transethnic meta-analysis. We observed an overlap of promoter and enhancer sequences at each locus and identified potential target genes. These data not only provided further support for the fine-mapping results but also revealed additional insights into the aetiology of glycaemic traits. UCSC Genome Browser images of each locus are provided in ESM Fig. 3. The results of our in silico functional annotations are summarised in ESM Table 7.

### Secondary associations at known glycaemic trait loci

To identify additional independent association signals at significant loci, conditional analyses were performed. Results of these analyses and population-specific associations are shown in Table 4. For transethnic conditional meta-analyses, ten fasting glucose loci and two fasting insulin loci were analysed. Independent secondary associations were identified at two fasting glucose loci (*G6PC2*-rs477224 and *GCK-*rs2908286). The second round of conditional analyses did not identify significant tertiary signals. Bioinformatic follow-up of rs477224 suggested that the variant is positioned within a pancreatic islet enhancer. The rs2908290 variant was in weak linkage disequilibrium (AMR *r* ^2^ = 0.26, AFR *r* ^2^ = 0.23) with a variant, rs2971677, predicted to alter splicing efficiency of *GCK*.

**Table 4:** Independent secondary signals at known fasting glucose and fasting insulin loci

Locus	Secondary SNP^a^	Frequency of coded (C) allele for secondary SNP					Effect of coded (C) allele for secondary SNP						*p* value^b^	Primary SNP^c^	LD *r* ^2d^	Cond. *p* value (second./primary)^e^
		C/N	TE	AA	H/L	AI/AN	ASN	TE	AA	H/L	AI/AN	ASN				
Transethnic meta-analysis fasting glucose																
*G6PC2*	rs477224	A/G	0.575	0.486	0.645	0.659	0.820	−0.036 (0.005)	−0.034 (0.007)***	−0.042 (0.007)***	0.035 (0.042)	−0.006 (0.035)	3 × 10^−14^	rs560887	<0.1	2 × 10^−5^/5 × 10^−26^
*GCK*	rs2908290	A/G	0.450	0.534	0.388	0.367	0.427	0.040 (0.005)	0.043 (0.007)***	0.038 (0.006)***	−0.009 (0.041)	0.058 (0.027)*	10 × 10^−18^	rs2908286	<0.1	2 × 10^−8^/6 × 10^−16^
Population-specific AA fasting glucose																
*G6PC2*	rs77719485	A/C	0.976	0.973	0.996	0.995	–	0.138 (0.020)	0.143 (0.022)***	0.115 (0.054)	−0.046 (0.283)	–	6x10^−11^	rs560887	<0.1	2 × 10^−6^/5 × 10^−7^

Sequential conditional analysis was performed on ten fasting glucose and two fasting insulin loci

In the AA fasting glucose analysis, rs77719485 was the most significant SNP in the locus and rs560887 was the second most significant. AA effects for rs560887 are shown in Table 3

^a^Lead SNP from conditional analysis reaching locus-specific significance

^b^ *p* value from the secondary SNP not adjusted for the primary SNP

^c^Lead SNP from primary (unconditional) analysis

^d^LD r^2^ between primary and secondary SNP

^e^ *p* values from conditional analysis

*p < 0.05 and ***p < 0.001 for race/ethnic-specific analyses

LD, linkage disequilibrium

To identify population-specific loci, we conducted separate conditional analyses for significant loci in the primary H/L (*GCKR*-rs1260326, *G6PC2*-rs560887, *SLC2A2-*rs1280, *DGKB*-rs1005256, *GCK*-rs1799884, *FADS3*-rs12577276, *MTNR1B*-rs10830963, *C2CD4A*-rs7167881), AA (*G6PC2*-rs77719485, *GCK*-rs2908286, *CRY2*-rs117493014, *MADD*-rs77082299, *ADCY5*-rs11708067, *MTNR1B*-rs10830963) and Asian populations (*GLIS3-*rs4395942). A population-specific variant was detected in the AA analysis of the *G6PC2* locus. The lead fasting glucose SNP, rs77719485, is less frequent in AA population (MAF 2.4%) and rare or monomorphic in the other populations (MAF 0.4% in H/L). Like the transethnic lead SNP, rs560887, bioinformatic follow-up suggested that rs77719485 may affect splicing efficiency for exon 4 for *G6PC2*.

### Association testing outside of glycaemic trait fine-mapping regions to identify potential novel variants

**Fig. 3:**
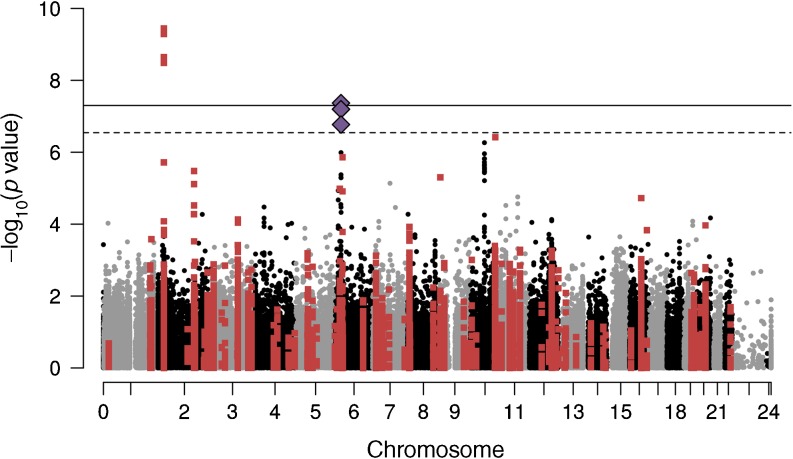
Fasting insulin association *p* values for each Metabochip variant from the transethnic meta-analysis in model without BMI. The –log_10_ of *p* values for each SNP on the Metabochip is plotted against chromosomal positions. Grey and black circles, SNPs alternating by chromosome; red squares, SNPs in previously reported glycaemic trait loci (within 1 Mb of index SNP *n* = 28,580); blue diamonds, novel SNP associations reaching Metabochip-wide significance (all are in the *SLC17A2* locus); solid line, threshold for Metabochip-wide significance (0.05/174,898 = 2.9 × 10^−7^); dashed line, threshold for genome-wide significance α = 5.0 × 10^−8^

In secondary analyses, we conducted a Metabochip-wide scan to identify potential novel or pleiotropic variants, given that the chip included variants with suggestive signals in established loci for many known metabolic traits. Models were run with and without BMI as a covariate (ESM Table 8, ESM Figs 4,5). Using the Bonferroni significance threshold (0.05/182,055 = 2.7 × 10^−7^), we identified one novel association for fasting insulin (rs75862513, *p* = 4.3 × 10^−8^, Fig. 3) at the *SLC17A2* locus previously associated with height and blood pressure [27, 28]. After BMI adjustment (ESM Fig. 5), the association was attenuated suggesting that the effects may be mediated by BMI.

## Discussion

In this large multiethnic study population of close to 30,000 participants, we used transethnic fine-mapping to narrow the list of putative causal variants for eleven glycaemic trait loci. On average, we observed a 72% reduction in the number of candidate SNPs, before bioinformatic follow-up. We further demonstrated that many of the genetic variants associated with glycaemic traits likely exert their effects through regulatory mechanisms (splicing or enhancer activity), and provide detailed annotations for subsequent laboratory follow-up. These regulatory annotations provide putative targets for laboratory follow-up (e.g. genome editing) and important insights into strong targets for future therapeutic interventions. For example, this study found that most of the implicated enhancer elements were binding sites for the transcription factor *FOXA2* in pancreatic islets, and previous studies have suggested that differential expression of *FOXA2* is a genetic determinant of fasting glucose levels, as well as type 2 diabetes risk [29, 30]. Like the previous European Metabochip analysis, we found that rs6113722, which is positioned within a lncRNA adjacent to *FOXA2*, was associated (*p* = 3.2 × 10^−8^) with fasting glucose. As such, expression levels of *FOXA2* could be a particularly important regulator of glucose homeostasis and a putative target for genome editing. Although the clinical application of genome editing is in its infancy, in vivo studies have already demonstrated the utility of the CRISPR/Cas9 technique. For example, to mimic observations of the naturally occurring loss-of-function mutation in the gene encoding LDL receptor antagonist PCSK9, a previous study in mice used CRISPR/Cas9 vectors to decrease PCSK9 protein levels, which resulted in increased hepatic LDL receptor levels, and a subsequent decrease in blood cholesterol levels [31]. Identification of key targets, such as *FOXA2*, and potential regulatory elements of these targets for laboratory follow-up is a critical first step in the translation of GWAS findings.

Analysis of known glycaemic trait loci in this diverse population study suggests the genetic determinants of glycaemic trait levels are likely to be similar across populations. In comparison with previous glycaemic trait studies conducted in diverse populations [7, 32], the replication of effects across populations is more extensive, likely due to the size of this study population. Although most of the loci in the European study were generalisable across populations, this study exemplifies the notion that analysis in diverse populations can refine known loci as well as help in the discovery of novel, sometimes population-specific, associations. For instance, in addition to the well-established splice variant rs560887 that has been robustly associated with fasting glucose, transethnic meta-analysis of the *G6PC2* locus identified an additional signal that may implicate regulatory functionality in glycaemia-related tissues. At this same locus, an AA-specific variant, rs77719485, was found to be strongly associated with fasting glucose and, like rs560887 [33], is predicted to affect splicing efficiency. By expanding our analysis to the entire Metabochip, we discovered strong associations with *SLC17A2*, that were not previously reported by the Metabochip analysis carried out by Scott et al [11] in Europeans. rs75862513 is a relatively rare variant that appears to be monomorphic in Europeans and was most frequent in the Asian (MAF = 0.04-A) and H/L (MAF = 0.001-A) populations in this study. If replicated in an independent dataset, this finding may represent a new locus not previously detected in European- or AA-specific analyses. These examples illustrate the power of transethnic analysis for locus refinement and novel discovery.

Strengths of this study include the large study size, high-density genotyping and representation of multiple diverse populations. In light of the heavy burden of hyperglycaemia in H/L and AA populations, this study begins to address the major gap in knowledge related to the genetic architecture of glycaemic traits in understudied American minority populations. The large study population, combined with new annotation resources, allowed transethnic fine-mapping and prediction of regulatory elements. However, there were several limitations that should be noted. Although this study included populations from four major racial/ethnic groups, the greatest proportions of participants were H/L and AA*.* As such, this study was limited in its ability to detect associations with more prominent effects in Asian populations [34, 35]*.* We also acknowledge that fine-mapping approaches only serve as an initial step in determining the underlying causal variant(s) driving association signals by prioritising likely causal candidates for more onerous laboratory follow-up. To further meet this objective, functional elements and variants were identified using bioinformatics databases. However, given that the functional evidence detected by these datasets is incomplete, future functional studies are critical in determining the underlying causal variants. That being said, the combination of fine-mapping with bioinformatics data is particularly useful for reducing both the physical genomic regions of interest and prioritising candidates for molecular characterisation. Furthermore, the in silico approaches help to provide richer inferences regarding the biological mechanisms modulating fasting glucose and insulin levels. As such, fine-mapping is an essential step in functional interpretation of GWAS signals because laboratory follow-up of all possible variants in GWAS loci is prohibitively expensive and time-intensive.

This transethnic study comprehensively fine-mapped known common variants associated with concentrations of fasting glucose and insulin. Genomic regions harbouring known risk variants were refined, novel functional candidates were proposed, new independent signals in previously fasting glucose-implicated genes were identified and one novel locus was discovered. Thus, these results suggest that transethnic meta-analysis can help in transforming GWAS results into new biological insight.

## Electronic supplementary material

ESM (PDF 4624 kb)

ESM Tables (XLSX 71 kb)

## Abbreviations

AA: African ancestry
AFR: African ancestry (1000 Genomes Super Population Code)
AI/AN: American Indian/Alaskan Native
AMR: Admixed American ancestry (1000 Genomes Super Population Code)
ARIC: Atherosclerosis Risk in Communities
ASN: Asian and Pacific Islander
CARDIA: Coronary Artery Risk Development in Young Adults
CEU: Utah Residents (CEPH) with Northern and Western European Ancestry (HapMap Population Code)
EUR: European ancestry (1000 Genomes Super Population Code)
GWAS: Genome-wide association studies
HCHS/SOL: Hispanic Community Health Study/Study of Latinos
H/L: Hispanic/Latino
MAF: Minor allele frequency
MAGIC: Meta-Analyses of Glucose and Insulin-related traits
MEC: The Multiethnic Cohort
NHGRI: National Human Genome Research Institute
PAGE: Population Architecture using Genetic Epidemiology
SHARe: WHI SNP Health Association Resource
WHI: Women’s Health Initiative
