# Supplementary material for: Transethnic insight into the genetics of glycaemic traits: fine-mapping results from the Population Architecture using Genomics and Epidemiology (PAGE) consortium
Source: Diabetologia. 2017 Sep 13;60(12):2384–98. doi: 10.1007/s00125-017-4405-1 (PMC5918310; doi:10.1007/s00125-017-4405-1)
Supplement: Supplementary file 1 — (PDF 4624 kb) [file 125_2017_4405_MOESM1_ESM.pdf]

ESM Methods

Study population and trait measurement

The Atherosclerosis Risk in Communities Study (ARIC) is part of Causal Variants Across the Life Course (CALiCo) consortium of well characterised population-based studies in bi-racial populations (white and African American) with a central genotyping and resequencing core laboratory [1]. ARIC is a multi-center prospective investigation of atherosclerotic disease in a predominantly bi-racial population conducted in four U.S. communities [1]. ARIC recruited 15,792 individuals (of which 4,266 are African American) aged 45-64 years from four communities in Forsyth County, N.C., Jackson, M.S., Minneapolis, M.N., and Washington County, M.D. for a baseline examination beginning in 1987, with annual follow-up until 2012, and semi-annual follow-up after occurring by telephone to maintain contact and assess health status. The data used in this study were from the baseline visit. BMI was calculated from weight and height measurements. Current smoking was dichotomised as yes/no according to the question "Do you now smoke cigarettes?" All African American subjects in ARIC were genotyped on the Metabochip. A detailed study protocol is available on the ARIC study website (https://www2.cscc.unc.edu/aric/).

The Coronary Artery Risk Development in Young Adults (CARDIA) Study is part of the CALiCo consortium and is a study examining the development and determinants of clinical and subclinical cardiovascular disease and their risk factors [2]. This study recruited 5,115 white and black individuals between 18 and 30 years old (52% African American and 55% women) in Birmingham, AL; Chicago, IL; Minneapolis, MN; and Oakland, CA [3]. CARDIA cohort participants, born between 1955 and 1968, have been influenced substantially by the obesity epidemic at ages younger than participants in other established NHLBI cohorts. Data were collected on a variety of factors believed to be related to heart disease, including fasting glucose levels. Physical measurements (height and weight), as well as lifestyle factors such as such as smoking and other chemistries (e.g., insulin), were collected. Current smoking was dichotomised using the following questions at the baseline questionnaire: "have you ever smoked cigarettes regularly for at least 3 months? By regularly, we mean at least 5 cigarettes per week, almost every week?" Those who stated "no" were considered to be non-smokers, while those who stated "yes" were then asked whether they currently smoked cigarettes. Those who responded "yes" were considered to be current smokers and those who responded "no" were considered to be former smokers. All study participants provided written informed consent. Only the African American participants were genotyped on Metabochip.

The Hispanic Community Health Study / Study of Latinos (HCHS/SOL) is a population-based cohort study of 16,415 self-identified Hispanic/Latino individuals aged 18-74 years randomly selected from households in four U.S. field centers (Chicago, IL; Miami, FL; Bronx, NY; San Diego, CA) [4]. The cohort includes participants who self-identified as having Hispanic/Latino background, the largest groups being Central American (*n* = 1,730), Cuban (*n* = 2,348), Dominican (*n* = 1,460), Mexican (*n* = 6,471), Puerto-Rican (*n* = 2,728), and South American (*n* = 1,068). The baseline examination during 2008 and 2011 included a clinical visit with comprehensive biological, behavioral, and socio-demographic assessments. Smoking status was measured by self-report and categorised into three groups: current, former, and never smokers. Two questions were used: "Have you ever smoked at least 100 cigarettes in your entire life?" and "Do you now smoke daily, some days or not at all?" If participants had smoked at least 100 cigarettes in their entire life and reported smoking daily or some days, then they were considered current smokers; if participants had smoked at least 100 cigarettes in their entire life and did not report smoking daily or some days, then they were considered former smokers; and if participants had not smoked at least 100 cigarettes in their entire life, they were considered never smokers.

The Women's Health Initiative (WHI) is a prospective study investigating post-menopausal women's health [5]. A total of 161,808 women aged 50–79 years old were recruited from 40 U.S. clinical centers between 1993 and 1998. WHI consists of two parts: randomised clinical trials of hormone therapy, dietary modification, and calcium/Vitamin D supplementation, and an observational cohort study. Socio-demographic characteristics, lifestyle factors (e.g. smoking), medical history, medication use and physical measures of height and weight were collected at the baseline visit. Data on lifetime active and passive smoking were collected. Women were initially classified by active smoking status into current, former or never smokers (participants that had not smoked 100 cigarettes in their life). A subset of African American participants was genotyped through the WHI SNP Health Association Resource (SHARe). All African American, Hispanic/Latino, Asian, and Native American/American Indian individuals who provided informed consent to submit their genotype data to dbGaP were either directly genotyped on the Metabochip or had genome-wide data on the Affymetrix 6.0 array available to impute Metabochip SNPs.

The Multiethnic Cohort (MEC) is a population-based prospective cohort study of over 215,000 men and women in Hawaii and California aged 45-75 at baseline (1993-1996) and primarily of five ethnic/racial groups: African Americans, Native Hawaiians, Whites, Latinos, and Japanese

Americans [6]. MEC was funded by the National Cancer Institute in 1993 to examine lifestyle risk factors and genetic susceptibility to cancer. All eligible cohort members completed baseline and follow-up questionnaires. Each participant completed a mailed, epidemiologic self-administered questionnaire regarding demographic, dietary, and lifestyle traits. This questionnaire included history of daily cigarette smoking during the past two weeks, smoking duration, and a record of current medications. Subjects were selected for Metabochip genotyping based on availability of biomarker data or from a pool of controls for a study of type 2 diabetes.

The BioMe Biobank Program is an ongoing, prospective, hospital- and outpatient-based population research program operated by The Charles Bronfman Institute for Personalized Medicine (IPM) at Mount Sinai. Bio*Me* is an Electronic Medical Record (EMR)-linked biobank that integrates research data and clinical care information for consented patients at The Mount Sinai Medical Center, which serves diverse local communities of upper Manhattan with broad health disparities. Bio*Me* populations include 36% Hispanics, 25% AA, 30% EA, and 9% of other ancestry. Biobank operations are fully integrated in clinical care processes, including direct recruitment from clinical sites waiting areas and phlebotomy stations by dedicated Biobank recruiters independent of clinical care providers, prior to or following a clinician standard of care visit. Recruitment currently occurs at a broad spectrum of over 30 clinical care sites. Information on anthropometrics, demographics, and medication was derived from both participants' EMR and a medical history questionnaire given at baseline [7]. Smoking status was measured by self-reported based on the following two questions: "Have you ever smoked cigarettes?" and "If you have quit, what year did you quit?" Height and Weight data are from questionnaires submitted at the time of Biobank enrollment. For the current analyses, genotype and phenotype data was available on Hispanic/Latinos and individuals with African Ancestry. Samples were genotyped using the Illumnia HumanOmniExpress+ v1.1 and then imputed with Impute2 [8] to genotypes of the 1000 Genomes project [9].

IRB Statement All participants included in these analyses have given consent for genetic studies and data sharing.

Continuous BMI measurement In all CALiCo studies, and WHI, BMI was calculated from height and weight measured at time of study enrolment in a clinic setting. In WHI only, measurements collected 1 or 3 years after enrolment were substituted for 140 participants missing enrolment

height and/or weight. In MEC and Bio*Me*, self-reported height and weight were used to calculate baseline BMI.

Smoking status measurement Smoking status was harmonised across studies as current versus former/never. See PAGE cohort descriptions above for study-specific details on ascertainment.

Racial/ethnic grouping In all studies, self-reported racial/ethnic group was collected via epidemiological questionnaires at baseline.

Glucose/insulin measurement Analyses were performed for fasting glucose (mmol/l) and natural log transformed fasting insulin (pmol/l). Individuals were excluded from the analysis if they were on diabetes treatment (oral or insulin), had a fasting plasma glucose equal to or greater than 7 mmol/l (126mg/dl), or fasting status for less than 8 hours. Individuals with BMI<16.5 kg/m^2 and BMI>70 kg/m^2 were also excluded with the assumption that these extremes could be attributable to data coding errors, an underlying illness, or possibly to a familial syndrome. In Bio*Me* , oral type 2 diabetes medications used for exclusion included Acetohexamide, Tolazamide, Chlorpropamide, Glipizide, Glyburide, Glimepiride, Repaglinide, Nateglinide, Metformin, Rosiglitazone, Pioglitazone, Troglitazone, Acarbose, Miglitol, Sitagliptin, and Exenatide.

Genotyping and quality control

DNA Extraction In the MEC and WHI, DNA was purified from buffy coat samples. A subset of MEC DNA samples were whole-genome amplified by Molecular Staging Inc. following their standard protocol. For CALiCo and Bio*Me* studies, DNA was extracted from blood samples drawn at baseline.

Genotyping Study specific details are summarised in ESM Table 1. Genotyping was performed using the Metabochip, a custom Illumina iSelect genotyping array. The Metabochip includes nearly 200,000 SNP markers and was designed to cost-effectively analyse putative association signals identified through GWAS meta-analyses of many obesity-related metabolic and cardiovascular traits and to fine-map established loci. Metabochip SNPs were selected from the catalogs developed by the International HapMap and 1000 Genomes Projects. More than 122,000 SNPs were included to fine-map 257 GWAS loci of 23 traits. The boundaries around each GWAS index SNP were determined by identifying all SNPs with $r^2 \geq 0.5$ with the index SNP, and then expanding the initial boundaries by 0.02cM in either direction using the HapMap-based genetic map. SNPs were

excluded if (a) the Illumina design score was <0.5 or (b) there were SNPs within 15bp in both directions of the SNP of interest with allele frequency of >0.02 among Europeans (CEU). SNPs annotated as nonsynonymous, essential splice site, or stop codon were included regardless of allele frequency, design score, or nearby SNPs in the primer.

Samples were genotyped at the Human Genetics Center of the University of Texas-Houston (ARIC and CARDIA), the University of Southern California Epigenome Center (MEC), the Genomics Core Facility of the Icahn Institute for Genomics and Multiscale Biology, Icahn School of Medicine at Mount Sinai (BioMe) and the Translational Genomics Research Institute (WHI). Each center genotyped the same 90 HapMap YRI (Yoruba in Ibadan, Nigeria) samples to facilitate cross-study quality control (QC), as well as 2-3% study-specific blinded replicates to assess genotyping quality. Genotypes were called separately for each study using GenomeStudio with the GenCall 2.0 algorithm. Samples were called using study-specific cluster definitions (based on samples with call rate >95%, ARIC, CARDIA, MEC, WHI, Bio*Me*) and kept in the analysis if call rate was >95%. We excluded SNPs with GenTrain score <0.6 (ARIC, CARDIA, MEC, WHI), Proper info score ≥ 0.4 (Bio*Me*), cluster separation score <0.4, call rate <0.95, and Hardy-Weinberg Equilibrium p value < 1×10^{-6}. We utilised the common 90 YRI samples and excluded any SNP that had more than 1 Mendelian error (in 30 YRI trios), any SNP that had more than two replication errors with discordant calls when comparisons were made across studies in 90 YRI samples, and any SNP that had more than three discordant calls for 90 YRI genotyped in PAGE versus the HapMap database. SNPs were excluded from the meta-analyses if they were present in less than three studies.

Replication and Fine-mapping of known glycaemic trait loci

For replication of index SNPs from GWAS, we used a nominal significance level (p value = 0.05). For transethnic signal fine-mapping we used the locus-specific p value (ranging from $\alpha = 1.41 \times 10^{-5}$ to $\alpha = 4.1 \times 10^{-4}$), which is 0.05 divided by the number of variants passing quality control at each locus.

To fine-map previously identified loci, we investigated the patterns of association at 15 known fasting glucose and fasting insulin loci using meta-analysis results from 13,613, and 2,406 SNPs genotyped on the Metabochip in up to 26,760, and 22,674 individuals for fasting glucose, and fasting insulin, respectively. At loci that exhibited evidence of regional significance (0.05/# of SNPs in region), we performed a series of sequential conditional analyses adding the most significant

lead SNP into the regression model as an additional covariate and testing all remaining regional SNPs not already in the model as a covariate for association. Sequential conditional analyses were performed adding in lead SNPs to the model until the strongest SNP association showed a conditional p value > than the regional significance level.

Regional plots for each locus were created showing data publicly available from the Scott et al European Metabochip analysis and using both the European lead SNP and the new lead SNPs from the trans-ethnic meta-analysis or from one of the population specific analyses. If the lead SNP from the current analysis was not present in the publicly available European Metabochip results a suitable proxy ($r^2 > 0.8$) was used. The plots were generated on the LocusZoom web-based plotting software using LD information from the 1000 Genomes Project in the most representative population European (hg19/Mar2012EUR data), AA (hg19/ Mar2012AFR data), H/L (hg19/Mar2012AMR data).

Strategy for selecting novel associations

The Metabochip selected genotyping content for type 2 diabetes, 2 hour glucose, glycated hemoglobin, fasting glucose, fasting insulin, myocardial infarction and coronary artery disease, high-density lipoprotein/low-density lipoprotein /triglycerides/total cholesterol, BMI, waist to hip ratio, body fat percentage, height, waist circumference, diastolic/systolic blood pressure, QT interval, mean platelet volume, platelet count, and white blood cell count. Given that Metabochip SNPs were included specifically due to prior evidence for association with these traits, the Metabochip-wide analyses were defined as testing for pleiotropy with any of these cardio, metabolic traits. Novel trait associations were subsequently investigated in the GWAS catalog to identify which trait(s) the locus was previously reported for. Metabochip-wide results were considered statistically significant if they reached a threshold of p value $\leq 2.5 \times 10^{-7}$ (0.05/196975), and were not in LD ($r^2 < 0.2$) or within 500 kb of a reported index SNP. Secondary independent or population-specific associations in the 15 fine-mapping regions are reported as being significant if they met region specific significance of 0.05/# SNPs in the locus.

Statistical analysis

All analyses were adjusted for age, sex, smoking status (current versus former/never), BMI, and ancestry principal components (PCs) in each study. Some studies also adjusted for center when applicable.

Each study performed race/ethnic specific analyses to test the association between continuous fasting glucose or natural log-transformed fasting insulin levels with genotypes or imputed dosages assuming an additive mode of inheritance. For studies of unrelated individuals, we applied multiple linear regression including age, sex, center site (as applicable), smoking status (current versus former/never), continuous BMI, and ancestry PCs (number varied by study) as model covariates. Like previous studies, primary analyses adjusted for BMI because it is a major risk factor for type 2 diabetes and is correlated with glycaemic traits. For sensitivity analysis, all models were also run without BMI as a covariate. Adjustment for smoking was decided *a priori* given the racial/ethnic differences in smoking patterns in the US and incident type 2 diabetes, as well as the association of cotinine with glycaemic related traits in non-diabetics [10]. HCHS/SOL has approximately 2000 related individuals, and a complex sampling design was used for recruitment. Therefore, in this study we employed the W-PS method (http://dlin.web.unc.edu/software/SUGEN/) by Lin et al, which is a weighted version of generalised estimation equations, to account for unequal inclusion probabilities and family relationships [11].

We combined SNP effect estimates and their standard errors across studies for H/L, AA, NA/AI and ASN by inverse-variance weighted fixed effects meta-analysis using METAL [12]. Results from these ethnic/race-specific meta-analyses are presented. Quantile-quantile plots for the Metabochip-wide analysis of fasting glucose and fasting insulin are shown in ESM Figure 4. Consistency between study/race-ethnicity effect size was assessed using the Q test (Chi-squared p value) and the I^2 metric, where low I^2 suggests little difference between-study/race-ethnicity variability. A two-stage conventional fixed-effects analysis approach was chosen to enable comparison of effects between races/ethnicities and because this strategy has been shown to provide a well-controlled type 1 error [13, 14]. Furthermore, I^2 metrics indicated little heterogeneity between race/ethnicities for the significant trans-ethnic results. Results for all analyses were reported as betas and standard errors (SE).

Functional Annotation

We interrogated each of the fine-mapped loci to determine if the identified non-coding variants were positioned within regulatory regions such as enhancers, promoters, insulators and/or silencers, which can potentially modulate transcript levels and thereby explain the underlying biology of the glucose/insulin association. To identify variants that overlapped putative regulatory elements we aligned a custom track with a list of correlated variants ($r^2 \geq 0.2$ in 1000 Genome

Project AMR and AFR) at each locus with several relevant tracks. Bedfiles were obtained using http://raggr.usc.edu/. Given that our traits of interest were glycaemic loci, we particularly utilised DNAseI hypersensitivity, histone modification and transcription factor occupancy data assayed in tissues and cell types derived from pancreatic, pancreatic islet, brain or other metabolically relevant tissues highlighted by expression patterns of strong candidate genes. By integrating the signal from several epigenomics tracks in the aforementioned tissues/cell types, a genomic element was annotated to be a putative enhancer if it showed enrichment for signal in DNASe hypersensitivity and H3K4me1/H3k27ac marks; as a promoter if it showed enrichment for signal in DNAse hypersensitivity and H3K4me3; as an insulator if it showed enrichment for signal for DNAse hypersensitivity, CTCF binding; and as a silencer if it showed enrichment for signal for DNAse Hypersensitivity and H3K9me3. A summary of the annotation for variants overlapping regulatory elements including noteworthy features like occupancy by key transcription factors and potentially interesting nearest genes candidates are provided in **ESM Table 19**. The epigenomics datasets were sourced from publicly available projects including ENCODE, Roadmap Epigenomics, and FANTOM5. Furthermore, to identify the motifs disrupted by putative risk alleles, we utilised Haploreg (v5) and the JASPAR motif database. To query GTEx eQTL data we utilised Haploreg (v5). Splice site misregulation was predicted using SPANR [15].

In addition to the use of epigenomic data, we also included summary functional scores from *in silico* prediction algorithms including RegulomeDB, the Combined Annotation Dependent Depletion (CADD) score, a PHRED-like score indicating deleteriousness of variant versus all other substitutions in genome. Additionally, we utilized the UCSC Genome Browser to interrogate TargetScan miRNA Regulatory Sites from 3’ UTR regions.

References

1. The ARIC investigators. (1989) The Atherosclerosis Risk in Communities (ARIC) Study: design and objectives. The ARIC investigators. Am J Epidemiol 129:687–702.

2. Friedman GD, Cutter GR, Donahue RP, et al. (1988) CARDIA: study design, recruitment, and some characteristics of the examined subjects. J Clin Epidemiol 41:1105–1116.

3. Hughes GH, Cutter G, Donahue R, et al. (1987) Recruitment in the Coronary Artery Disease Risk Development in Young Adults (Cardia) Study. Control Clin Trials 8:68S–73S.

4. Sorlie PD, Avilés-Santa LM, Wassertheil-Smoller S, et al. (2010) Design and implementation of the Hispanic Community Health Study/Study of Latinos. Ann Epidemiol 20:629–641.

5. The Women's Health Initiative Study Group. (1998) Design of the Women's Health Initiative clinical trial and observational study. Control Clin Trials 19:61–109.

6. Kolonel LN, Henderson BE, Hankin JH, et al. (2000) A multiethnic cohort in Hawaii and Los Angeles: baseline characteristics. Am J Epidemiol 151:346–357.

7. Gottesman O, Kuivaniemi H, Tromp G, et al. (2013) The Electronic Medical Records and Genomics (eMERGE) Network: past, present, and future. Genet Med 15:761–771.

8. Howie BN, Donnelly P, Marchini J (2009) A flexible and accurate genotype imputation method for the next generation of genome-wide association studies. PLoS Genet 5:e1000529. doi: 10.1371/journal.pgen.1000529

9. 1000 Genomes Project Consortium, Abecasis GR, Auton A, et al. (2012) An integrated map of genetic variation from 1,092 human genomes. Nature 491:56–65.

10. Liu R, Zheng Z, Du J, et al. (2016) Racial Disparity in the Associations of Cotinine with Insulin Secretion: Data from the National Health and Nutrition Examination Survey, 2007-2012. PLoS ONE 11:e0167260. doi: 10.1371/journal.pone.0167260

11. Lin D-Y, Tao R, Kalsbeek WD, et al. (2014) Genetic association analysis under complex survey sampling: the Hispanic Community Health Study/Study of Latinos. Am J Hum Genet 95:675–688.

12. Willer CJ, Li Y, Abecasis GR (2010) METAL: fast and efficient meta-analysis of genomewide association scans. Bioinformatics 26:2190–2191.

13. Hong J, Lunetta KL, Cupples LA, et al. (2016) Evaluation of a Two-Stage Approach in Trans-Ethnic Meta-Analysis in Genome-Wide Association Studies. Genet Epidemiol 40:284–292.

14. Franceschini N, Carty CL, Lu Y, et al. (2016) Variant Discovery and Fine Mapping of Genetic Loci Associated with Blood Pressure Traits in Hispanics and African Americans. PLoS ONE 11:e0164132. doi: 10.1371/journal.pone.0164132

15. Xiong HY, Alipanahi B, Lee LJ, et al. (2015) RNA splicing. The human splicing code reveals new insights into the genetic determinants of disease. Science 347:1254806.

16. Gao N, LeLay J, Vatamaniuk MZ, et al. (2008) Dynamic regulation of Pdx1 enhancers by Foxa1 and Foxa2 is essential for pancreas development. Genes Dev 22:3435–3448.

17. Sund NJ, Vatamaniuk MZ, Casey M, et al. (2001) Tissue-specific deletion of Foxa2 in pancreatic beta cells results in hyperinsulinemic hypoglycemia. Genes Dev 15:1706–1715.

18. Zou F, Chai HS, Younkin CS, et al. (2012) Brain expression genome-wide association study (eGWAS) identifies human disease-associated variants. PLoS Genet 8:e1002707.

19. GTEx Consortium (2015) Human genomics. The Genotype-Tissue Expression (GTEx) pilot analysis: multitissue gene regulation in humans. Science 348:648–660.

20. Lyssenko V, Nagorny CLF, Erdos MR, et al. (2009) Common variant in MTNR1B associated with increased risk of type 2 diabetes and impaired early insulin secretion. Nat Genet 41:82–88.

ESM Figure 1

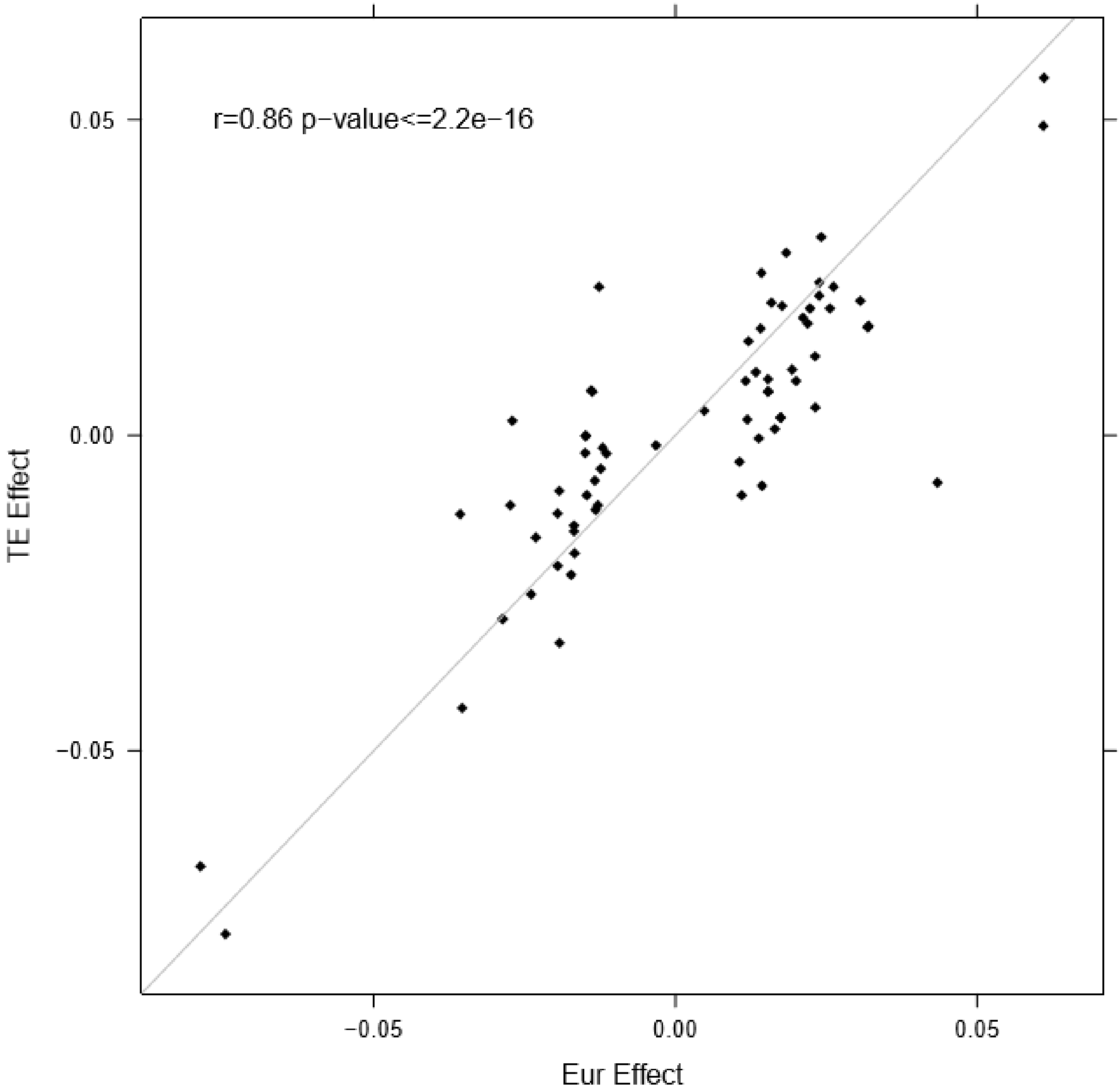

Comparison of effect estimates for index SNP associations for fasting glucose and insulin between trans-ethnic (TE) meta-analysis and published European (EUR) Metabochip. Results are shown for models adjusted for BMI. PAGE transethnic meta-analysis results from Metabochip are compared to the Scott et al study of European-descent MetaboChip results in up to 99,029 samples for fasting insulin and 118,881 samples for fasting glucose.

ESM Figure 2

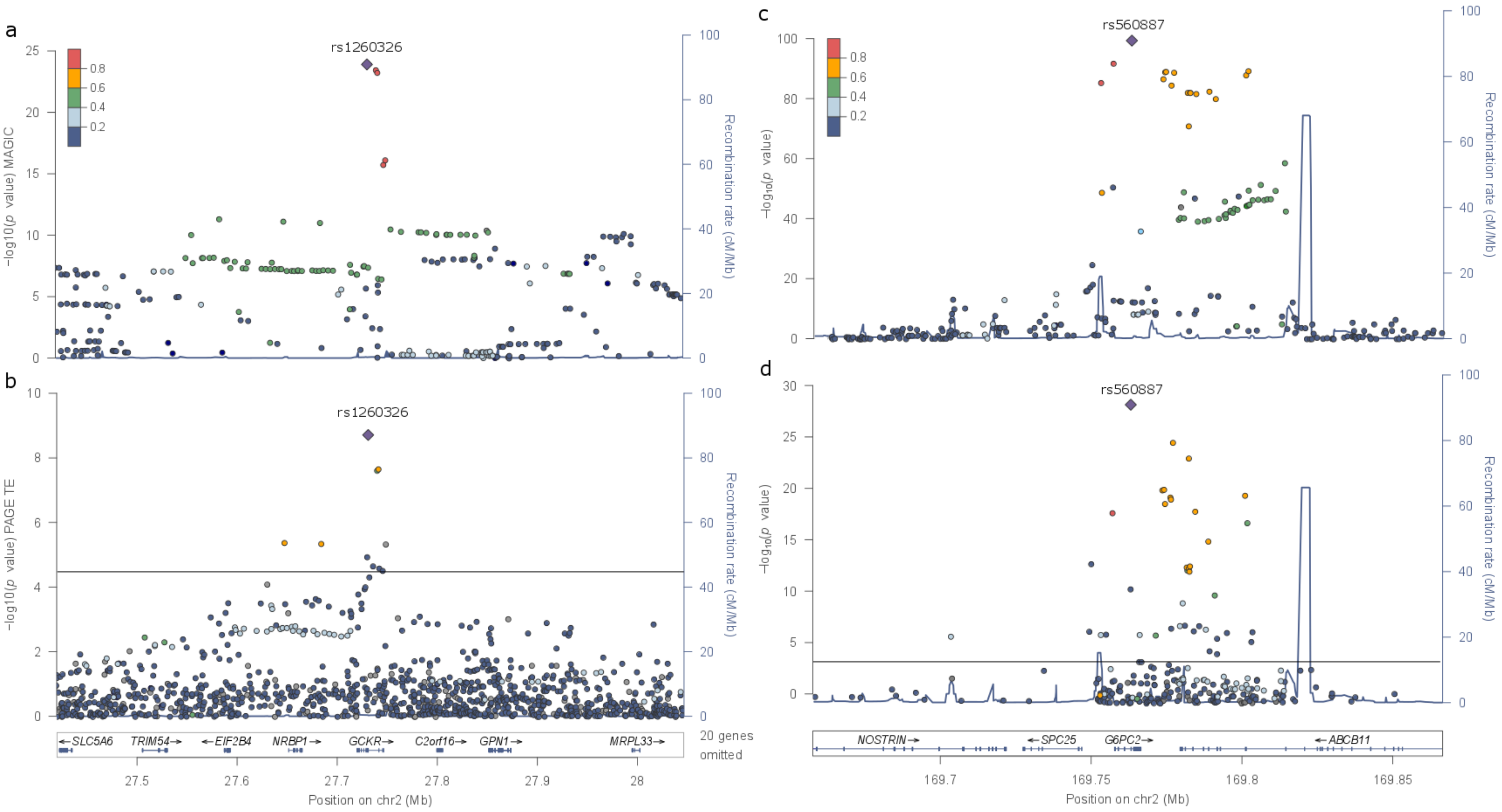

a
rs1260326
−log10(p value) MAGIC
Recombination rate (cM/Mb)
0.8
0.6
0.4
0.2
b
rs1260326
−log10(p value) PAGE TE
Recombination rate (cM/Mb)
SLC5A6
TRIM54
EIF2B4
NRBP1
GCKR
C2orf16
GPN1
MRPL33
20 genes omitted
27.5
27.6
27.7
27.8
27.9
28
Position on chr2 (Mb)
c
rs560887
−log10(p value)
Recombination rate (cM/Mb)
0.8
0.6
0.4
0.2
d
rs560887
−log10(p value)
Recombination rate (cM/Mb)
NOSTRIN
SPC25
G6PC2
ABCB11
169.7
169.75
169.8
169.85
Position on chr2 (Mb)

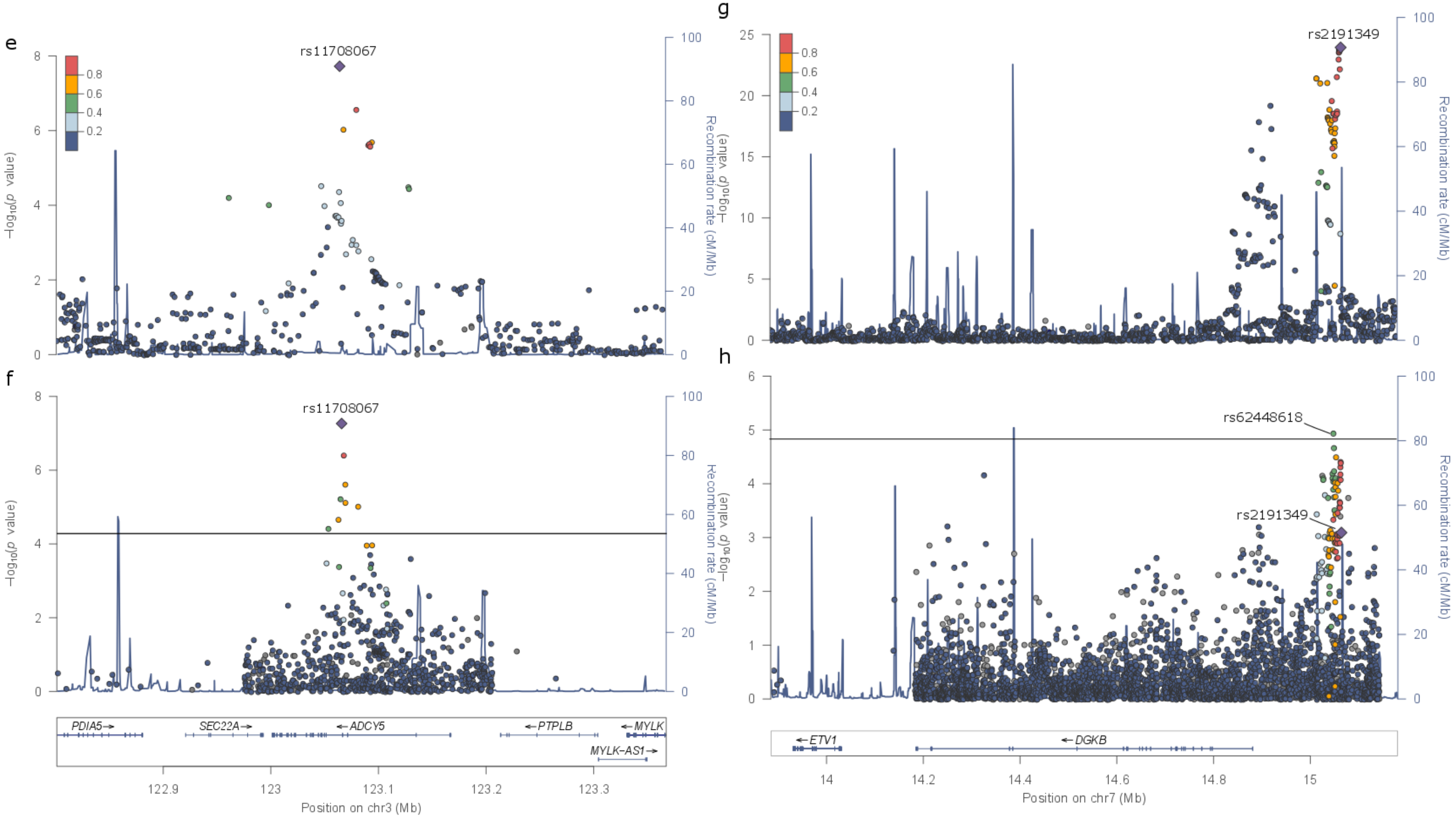

e
rs11708067
f
rs11708067
g
rs2191349
h
rs62448618
rs2191349
0.8
0.6
0.4
0.2
−log10(p value)
Recombination rate (cM/Mb)
PDIA5→
SEC22A→
←ADCY5
←PTPLB
←MYLK
MYLK-AS1→
122.9
123
123.1
123.2
123.3
Position on chr3 (Mb)
←ETV1
←DGKB
14
14.2
14.4
14.6
14.8
15
Position on chr7 (Mb)

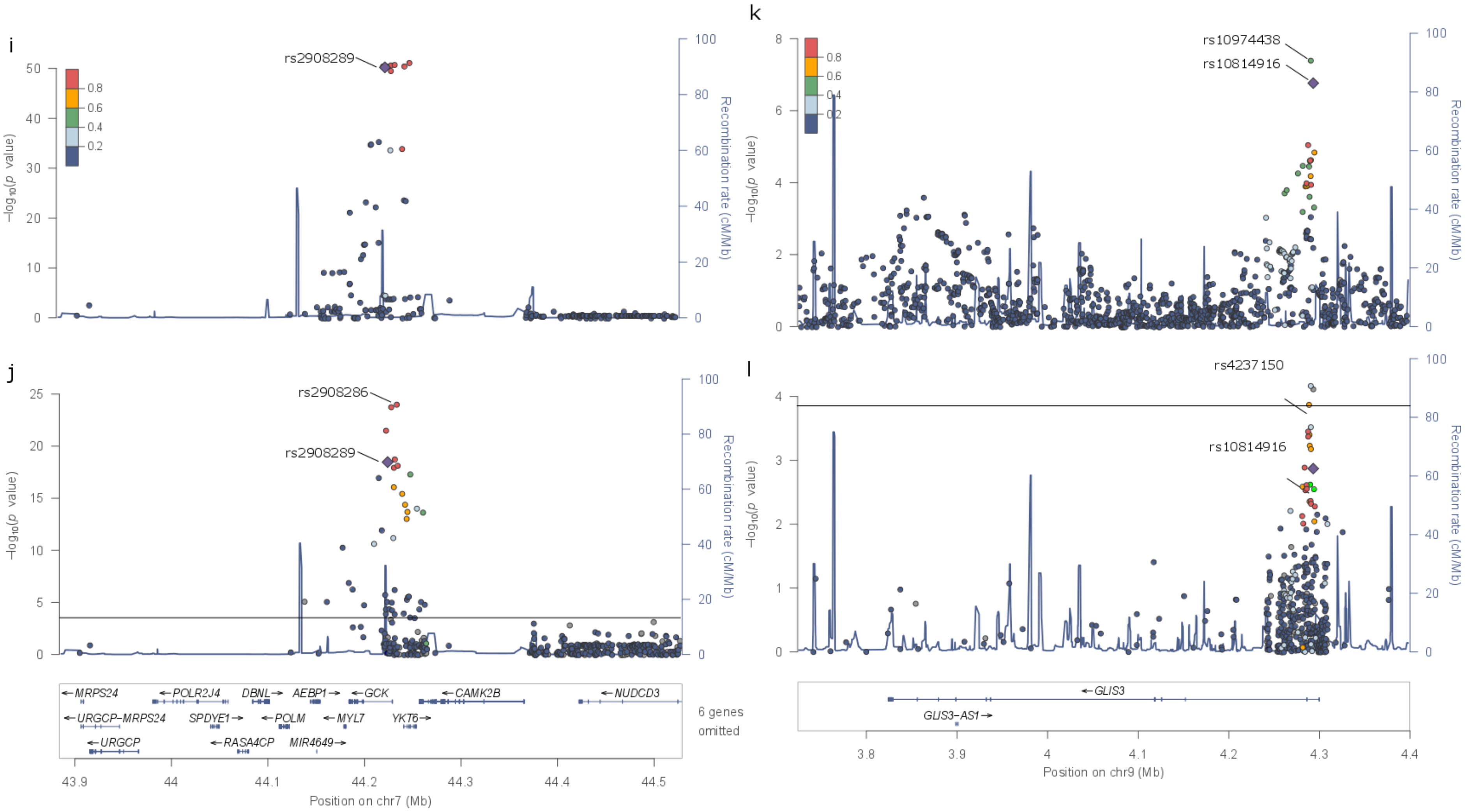

i
rs2908289
0.8
0.6
0.4
0.2
−log10(p value)
Recombination rate (cM/Mb)
j
rs2908286
rs2908289
MRPS24
POLR2J4
DBNL
AEBP1
GCK
CAMK2B
NUDCD3
URGCP-MRPS24
SPDYE1
POLM
MYL7
YKT6
URGCP
RASA4CP
MIR4649
6 genes omitted
Position on chr7 (Mb)
k
rs10974438
rs10814916
l
rs4237150
rs10814916
GLIS3
GLIS3-AS1
Position on chr9 (Mb)

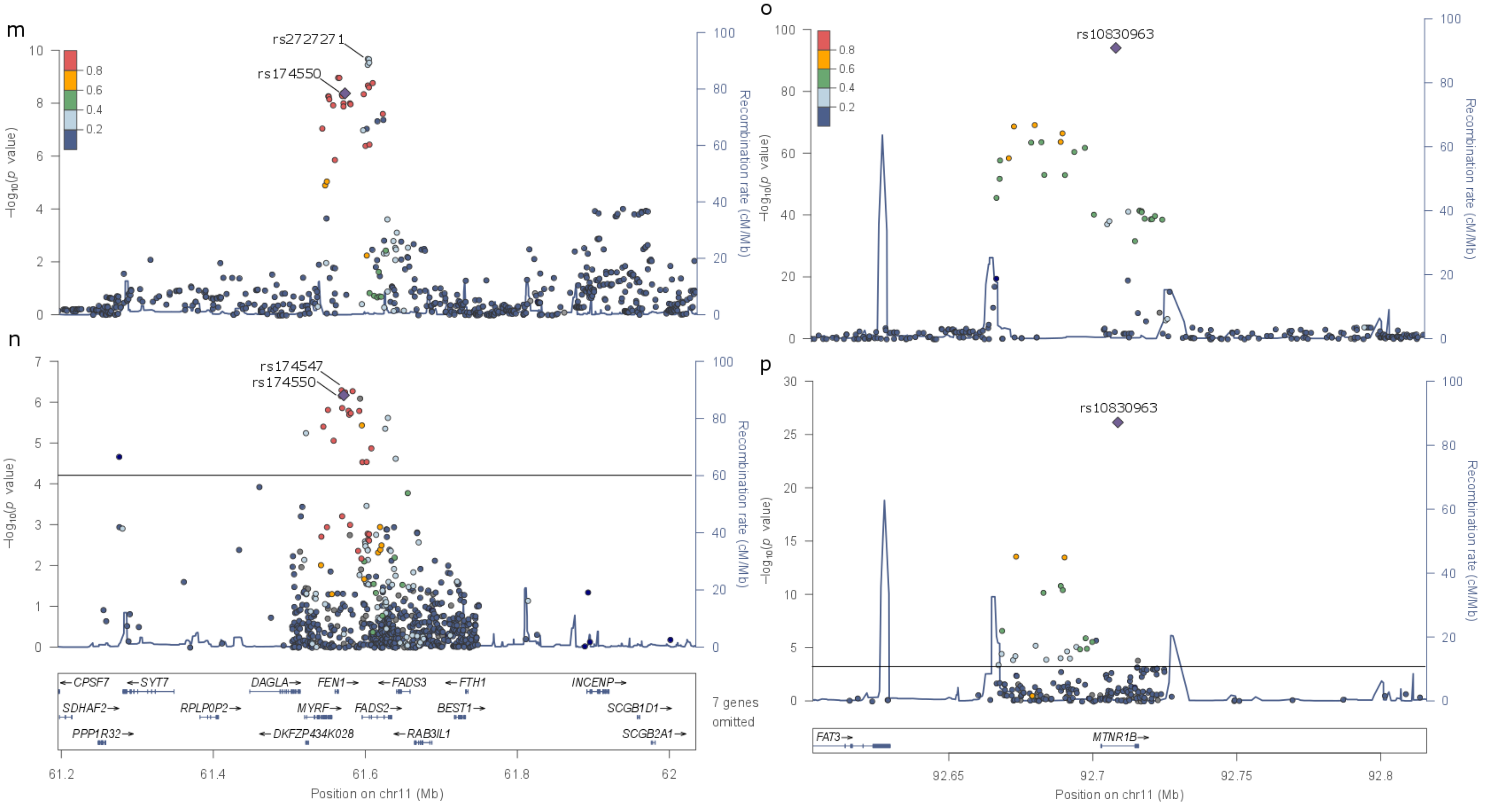

m
n
o
p
rs2727271
rs174550
rs174547
rs10830963
0.8
0.6
0.4
0.2
−log10(p value)
Recombination rate (cM/Mb)
CPSF7
SYT7
DAGLA
FEN1
FADS3
FTH1
INCENP
SDHAF2
RPLP0P2
MYRF
FADS2
BEST1
SCGB1D1
PPP1R32
DKFZP434K028
RAB3IL1
SCGB2A1
7 genes omitted
Position on chr11 (Mb)
FAT3
MTNR1B

q

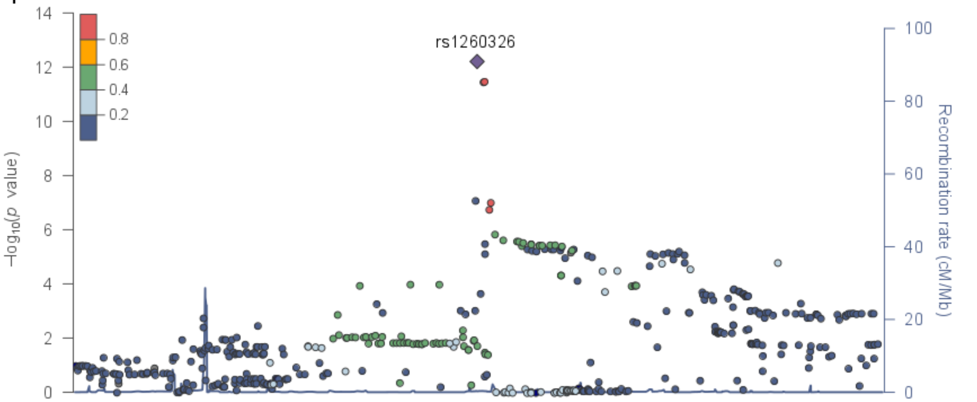

r

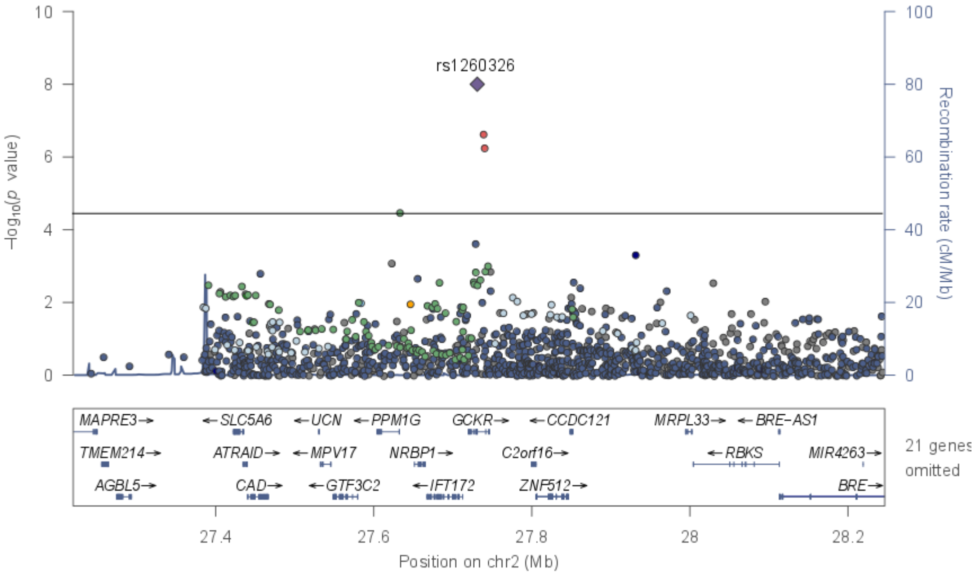

Regional plots for fine-mapping loci comparing Metabochip results for MAGIC European population (top) to PAGE transethnic meta-analysis (bottom). Each SNP in the 8 fasting glucose loci and 1 fasting insulin locus with evidence of fine-mapping is plotted with association results, -log10(*p* value), on the y-axis, and chromosomal position on the x-axis. Fasting glucose results are shown for: (a,b) *GCKR*, (c,d) *G6PC2*, (e,f) *ADCY5*, (g,h) *DGKB*, (i,j) *GCK*, (k,l) *GLIS3*, (m,n) *FADS2*, (o,p) *MTNR1B*. Fasting insulin results are shown for (q,r) *GCKR*. Not all SNPs used in the transethnic meta-analysis were present in the available MAGIC data (http://www.magicinvestigators.org/downloads/) due to mapping

issues [16]. SNPs not passing QC or outside of fine-mapping region were removed from the TE plots. The colour scale legend indicates linkage disequilibrium (r^2) between each fine-mapping SNP and the GWAS index SNPs (purple diamonds), which was calculated using 1000 Genomes Populations (CEU for MAGIC and AMR or AFR for PAGE). The population chosen for LD colouring in the transethnic meta-analysis was based on population-specific analysis results (choosing the population with the most significant underlying SNP associations, AMR for all except *GCKR* (b), and *ADCY5* (f). Solid line corresponds to locus specific significance threshold.

ESM Figure 3

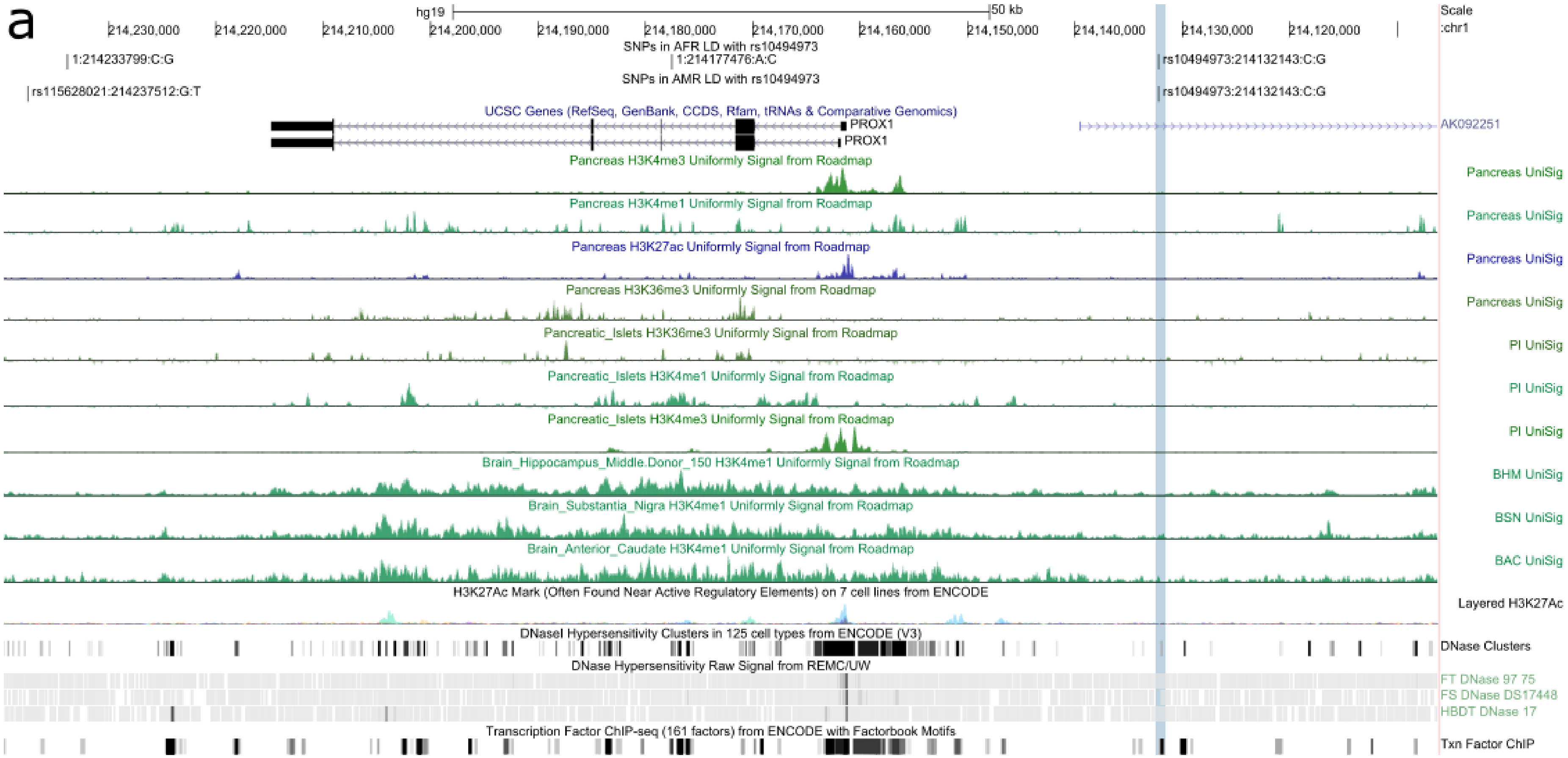

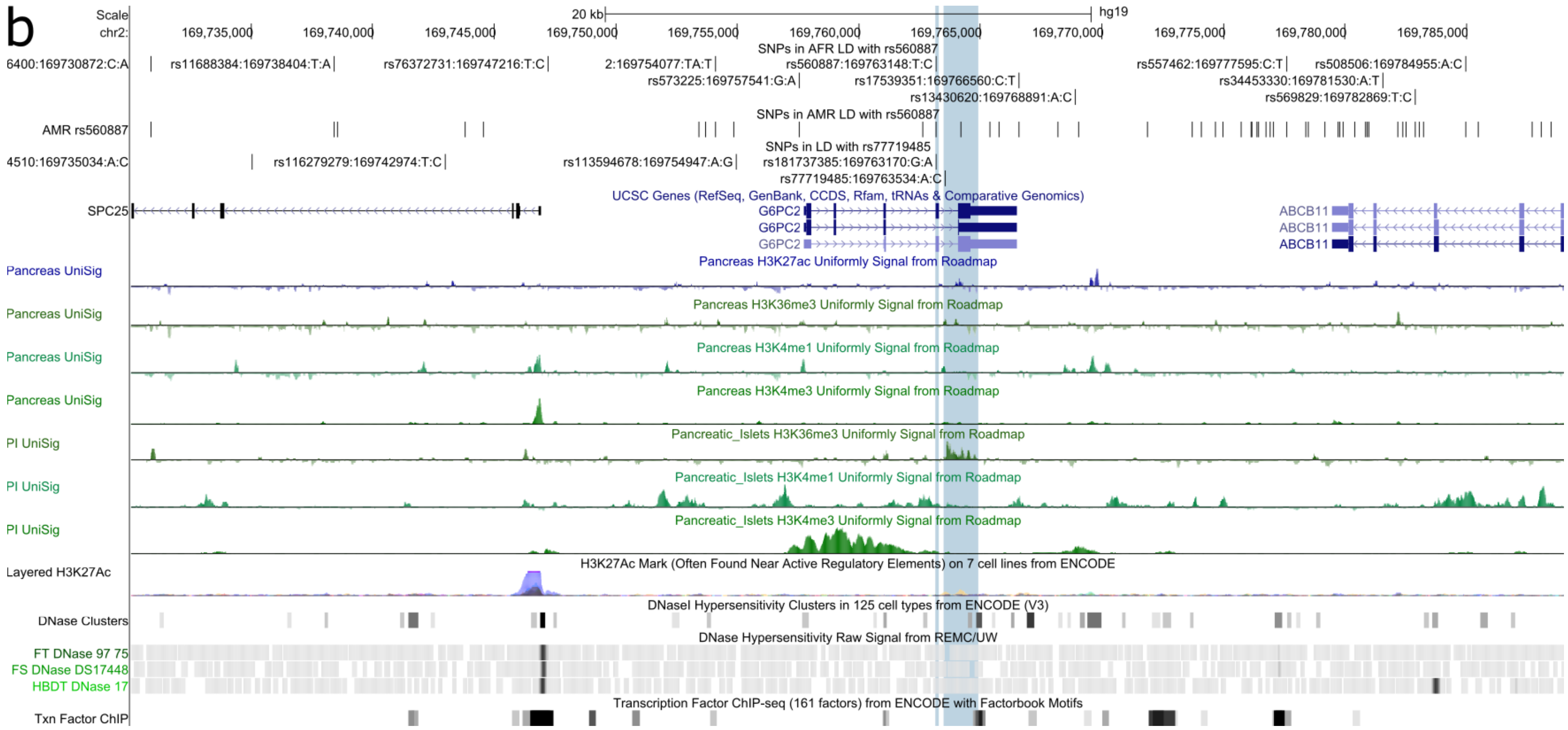
b
Scale
chr2:
20 kb
hg19
169,735,000
169,740,000
169,745,000
169,750,000
169,755,000
169,760,000
169,765,000
169,770,000
169,775,000
169,780,000
169,785,000
SNPs in AFR LD with rs560887
6400:169730872:C:A
rs11688384:169738404:T:A
rs76372731:169747216:T:C
2:169754077:TA:T
rs560887:169763148:T:C
rs557462:169777595:C:T
rs508506:169784955:A:C
rs573225:169757541:G:A
rs17539351:169766560:C:T
rs34453330:169781530:A:T
rs13430620:169768891:A:C
rs569829:169782869:T:C
SNPs in AMR LD with rs560887
AMR rs560887
SNPs in LD with rs77719485
4510:169735034:A:C
rs116279279:169742974:T:C
rs113594678:169754947:A:G
rs181737385:169763170:G:A
rs77719485:169763534:A:C
UCSC Genes (RefSeq, GenBank, CCDS, Rfam, tRNAs & Comparative Genomics)
SPC25
G6PC2
G6PC2
G6PC2
ABCB11
ABCB11
ABCB11
Pancreas UniSig
Pancreas H3K27ac Uniformly Signal from Roadmap
Pancreas UniSig
Pancreas H3K36me3 Uniformly Signal from Roadmap
Pancreas UniSig
Pancreas H3K4me1 Uniformly Signal from Roadmap
Pancreas UniSig
Pancreas H3K4me3 Uniformly Signal from Roadmap
PI UniSig
Pancreatic_Islets H3K36me3 Uniformly Signal from Roadmap
PI UniSig
Pancreatic_Islets H3K4me1 Uniformly Signal from Roadmap
PI UniSig
Pancreatic_Islets H3K4me3 Uniformly Signal from Roadmap
Layered H3K27Ac
H3K27Ac Mark (Often Found Near Active Regulatory Elements) on 7 cell lines from ENCODE
DNase Clusters
DNaseI Hypersensitivity Clusters in 125 cell types from ENCODE (V3)
DNase Hypersensitivity Raw Signal from REMC/UW
FT DNase 97 75
FS DNase DS17448
HBDT DNase 17
Transcription Factor ChIP-seq (161 factors) from ENCODE with Factorbook Motifs
Txn Factor ChIP

C

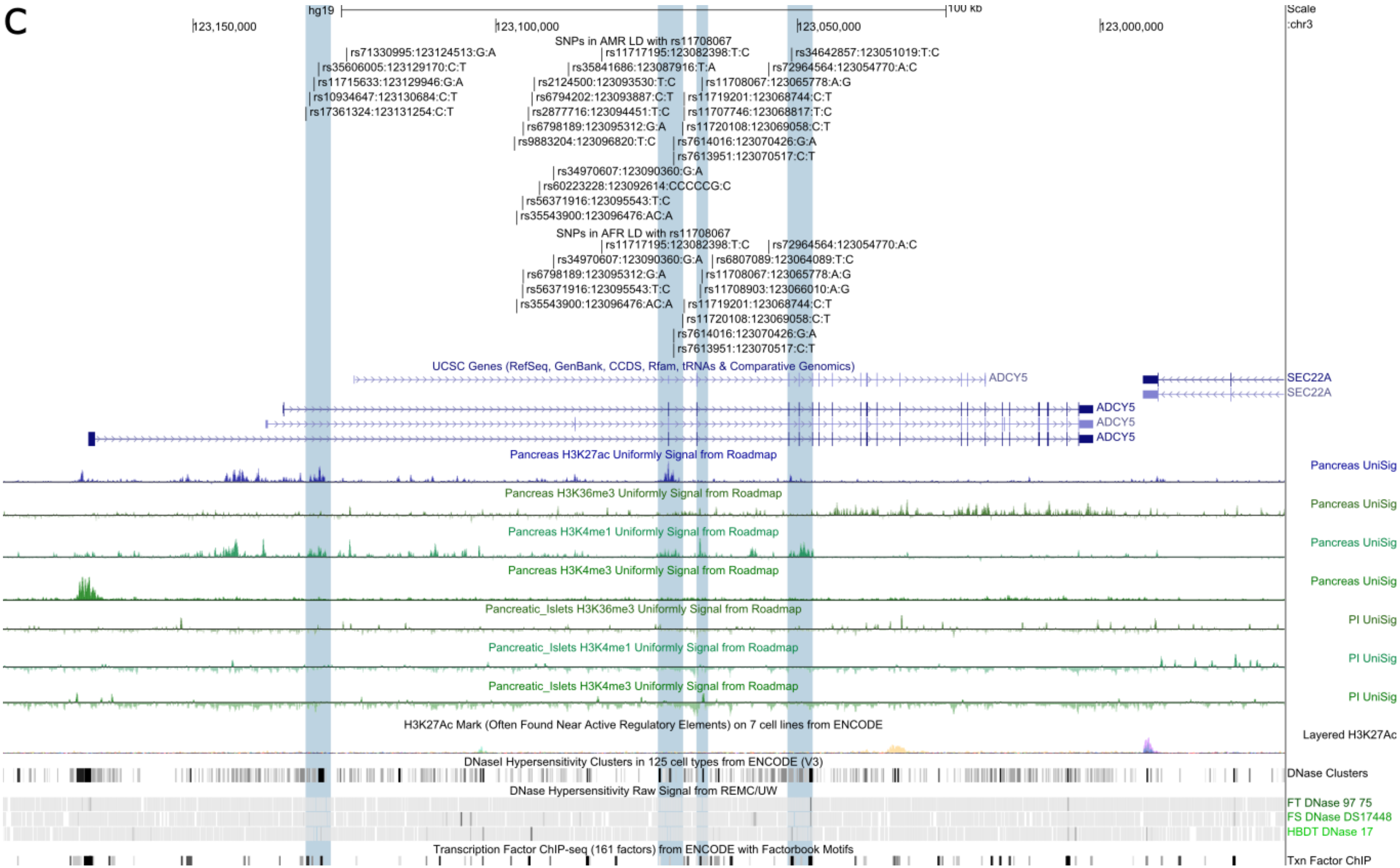

hg19
100 kb
Scale
:chr3
123,150,000
123,100,000
123,050,000
123,000,000
SNPs in AMR LD with rs11708067
rs71330995:123124513:G:A
rs35606005:123129170:C:T
rs11715633:123129946:G:A
rs10934647:123130684:C:T
rs17361324:123131254:C:T
rs11717195:123082398:T:C
rs35841686:123087916:T:A
rs2124500:123093530:T:C
rs6794202:123093887:C:T
rs2877716:123094451:T:C
rs6798189:123095312:G:A
rs9883204:123096820:T:C
rs34970607:123090360:G:A
rs60223228:123092614:CCCCCG:C
rs56371916:123095543:T:C
rs35543900:123096476:AC:A
rs34642857:123051019:T:C
rs72964564:123054770:A:C
rs11708067:123065778:A:G
rs11719201:123068744:C:T
rs11707746:123068817:T:C
rs11720108:123069058:C:T
rs7614016:123070426:G:A
rs7613951:123070517:C:T
SNPs in AFR LD with rs11708067
rs11717195:123082398:T:C
rs34970607:123090360:G:A
rs6798189:123095312:G:A
rs56371916:123095543:T:C
rs35543900:123096476:AC:A
rs72964564:123054770:A:C
rs6807089:123064089:T:C
rs11708067:123065778:A:G
rs11708903:123066010:A:G
rs11719201:123068744:C:T
rs11720108:123069058:C:T
rs7614016:123070426:G:A
rs7613951:123070517:C:T
UCSC Genes (RefSeq, GenBank, CCDS, Rfam, tRNAs & Comparative Genomics)
ADCY5
SEC22A
SEC22A
ADCY5
ADCY5
ADCY5
Pancreas H3K27ac Uniformly Signal from Roadmap
Pancreas UniSig
Pancreas H3K36me3 Uniformly Signal from Roadmap
Pancreas UniSig
Pancreas H3K4me1 Uniformly Signal from Roadmap
Pancreas UniSig
Pancreas H3K4me3 Uniformly Signal from Roadmap
Pancreas UniSig
Pancreatic_Islets H3K36me3 Uniformly Signal from Roadmap
PI UniSig
Pancreatic_Islets H3K4me1 Uniformly Signal from Roadmap
PI UniSig
Pancreatic_Islets H3K4me3 Uniformly Signal from Roadmap
PI UniSig
H3K27Ac Mark (Often Found Near Active Regulatory Elements) on 7 cell lines from ENCODE
Layered H3K27Ac
DNaseI Hypersensitivity Clusters in 125 cell types from ENCODE (V3)
DNase Clusters
DNase Hypersensitivity Raw Signal from REMC/UW
FT DNase 97 75
FS DNase DS17448
HBDT DNase 17
Transcription Factor ChIP-seq (161 factors) from ENCODE with Factorbook Motifs
Txn Factor ChIP

d

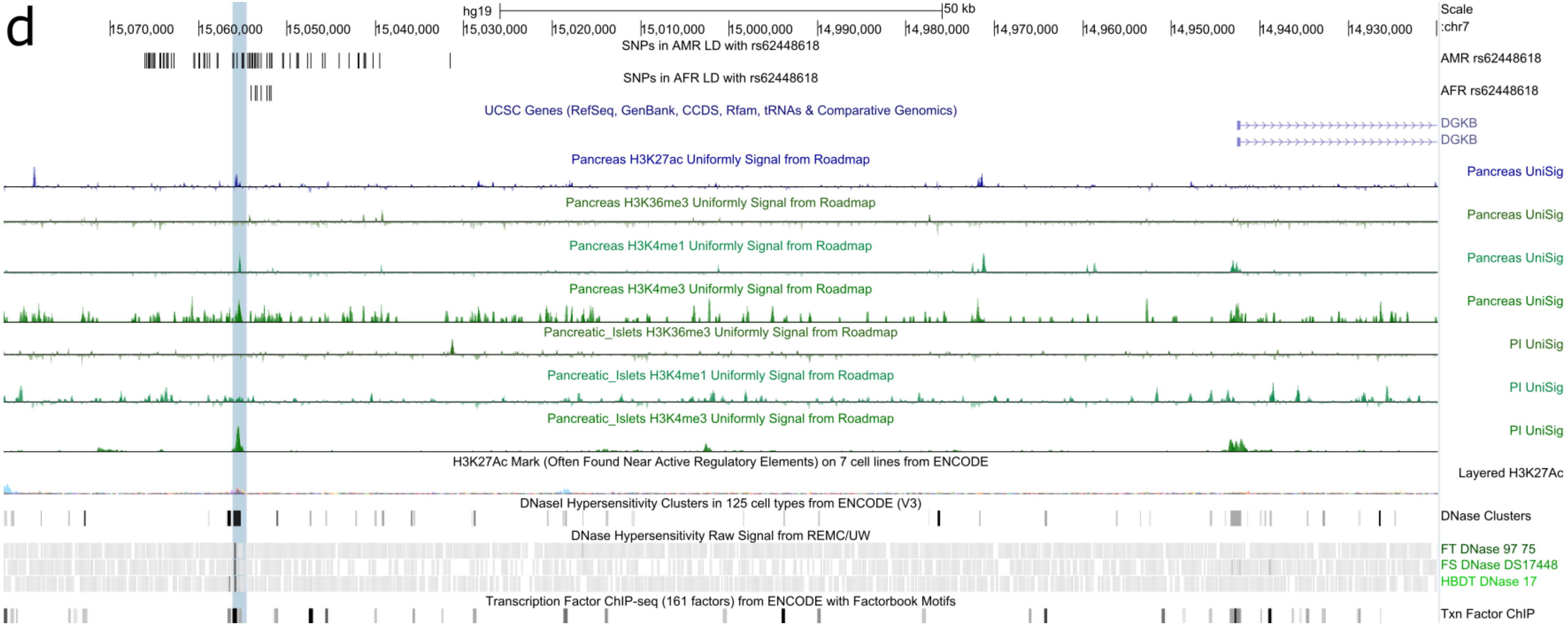
hg19
50 kb
Scale
chr7
15,070,000
15,060,000
15,050,000
15,040,000
15,030,000
15,020,000
15,010,000
15,000,000
14,990,000
14,980,000
14,970,000
14,960,000
14,950,000
14,940,000
14,930,000
SNPs in AMR LD with rs62448618
AMR rs62448618
SNPs in AFR LD with rs62448618
AFR rs62448618
UCSC Genes (RefSeq, GenBank, CCDS, Rfam, tRNAs & Comparative Genomics)
DGKB
DGKB
Pancreas H3K27ac Uniformly Signal from Roadmap
Pancreas UniSig
Pancreas H3K36me3 Uniformly Signal from Roadmap
Pancreas UniSig
Pancreas H3K4me1 Uniformly Signal from Roadmap
Pancreas UniSig
Pancreas H3K4me3 Uniformly Signal from Roadmap
Pancreas UniSig
Pancreatic_Islets H3K36me3 Uniformly Signal from Roadmap
PI UniSig
Pancreatic_Islets H3K4me1 Uniformly Signal from Roadmap
PI UniSig
Pancreatic_Islets H3K4me3 Uniformly Signal from Roadmap
PI UniSig
H3K27Ac Mark (Often Found Near Active Regulatory Elements) on 7 cell lines from ENCODE
Layered H3K27Ac
DNaseI Hypersensitivity Clusters in 125 cell types from ENCODE (V3)
DNase Clusters
DNase Hypersensitivity Raw Signal from REMC/UW
FT DNase 97 75
FS DNase DS17448
HBDT DNase 17
Transcription Factor ChIP-seq (161 factors) from ENCODE with Factorbook Motifs
Txn Factor ChIP

e

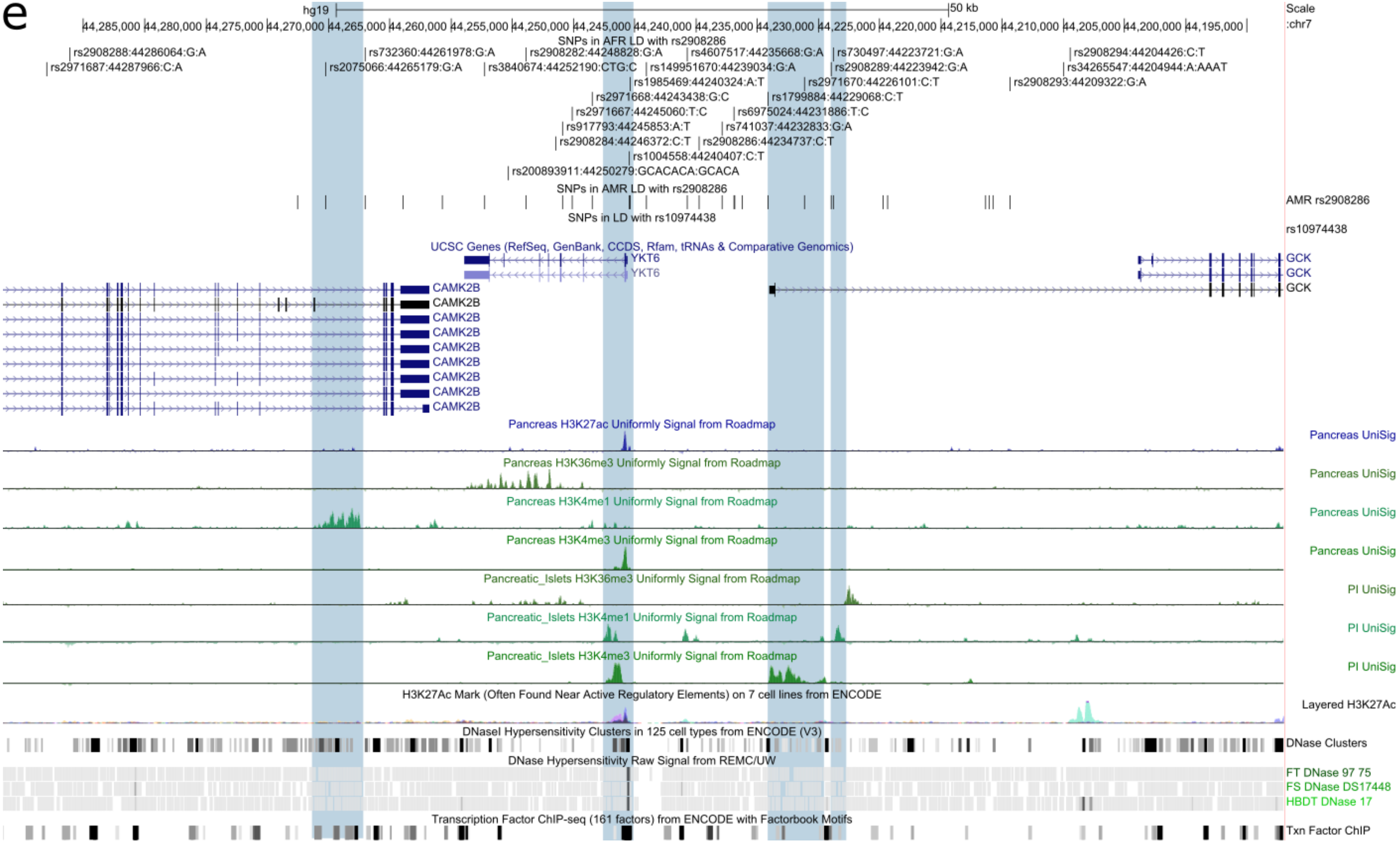

Scale
50 kb
hg19
chr7
44,285,000 44,280,000 44,275,000 44,270,000 44,265,000 44,260,000 44,255,000 44,250,000 44,245,000 44,240,000 44,235,000 44,230,000 44,225,000 44,220,000 44,215,000 44,210,000 44,205,000 44,200,000 44,195,000
SNPs in AFR LD with rs2908286
rs2908288:44286064:G:A
rs2971687:44287966:C:A
rs732360:44261978:G:A
rs2075066:44265179:G:A
rs2908282:44248828:G:A
rs3840674:44252190:CTG:C
rs4607517:44235668:G:A
rs149951670:44239034:G:A
rs730497:44223721:G:A
rs2908289:44223942:G:A
rs2908294:44204426:C:T
rs34265547:44204944:A:AAAT
rs1985469:44240324:A:T
rs2971670:44226101:C:T
rs2908293:44209322:G:A
rs2971668:44243438:G:C
rs1799884:44229068:C:T
rs2971667:44245060:T:C
rs6975024:44231886:T:C
rs917793:44245853:A:T
rs741037:44232833:G:A
rs2908284:44246372:C:T
rs2908286:44234737:C:T
rs1004558:44240407:C:T
rs200893911:44250279:GCACACA:GCACA
SNPs in AMR LD with rs2908286
AMR rs2908286
SNPs in LD with rs10974438
rs10974438
UCSC Genes (RefSeq, GenBank, CCDS, Rfam, tRNAs & Comparative Genomics)
YKT6
YKT6
GCK
GCK
GCK
CAMK2B
CAMK2B
CAMK2B
CAMK2B
CAMK2B
CAMK2B
CAMK2B
CAMK2B
CAMK2B
Pancreas H3K27ac Uniformly Signal from Roadmap
Pancreas UniSig
Pancreas H3K36me3 Uniformly Signal from Roadmap
Pancreas UniSig
Pancreas H3K4me1 Uniformly Signal from Roadmap
Pancreas UniSig
Pancreas H3K4me3 Uniformly Signal from Roadmap
Pancreas UniSig
Pancreatic_Islets H3K36me3 Uniformly Signal from Roadmap
PI UniSig
Pancreatic_Islets H3K4me1 Uniformly Signal from Roadmap
PI UniSig
Pancreatic_Islets H3K4me3 Uniformly Signal from Roadmap
PI UniSig
H3K27Ac Mark (Often Found Near Active Regulatory Elements) on 7 cell lines from ENCODE
Layered H3K27Ac
DNaseI Hypersensitivity Clusters in 125 cell types from ENCODE (V3)
DNase Clusters
DNase Hypersensitivity Raw Signal from REMC/UW
FT DNase 97 75
FS DNase DS17448
HBDT DNase 17
Transcription Factor ChIP-seq (161 factors) from ENCODE with Factorbook Motifs
Txn Factor ChIP

f

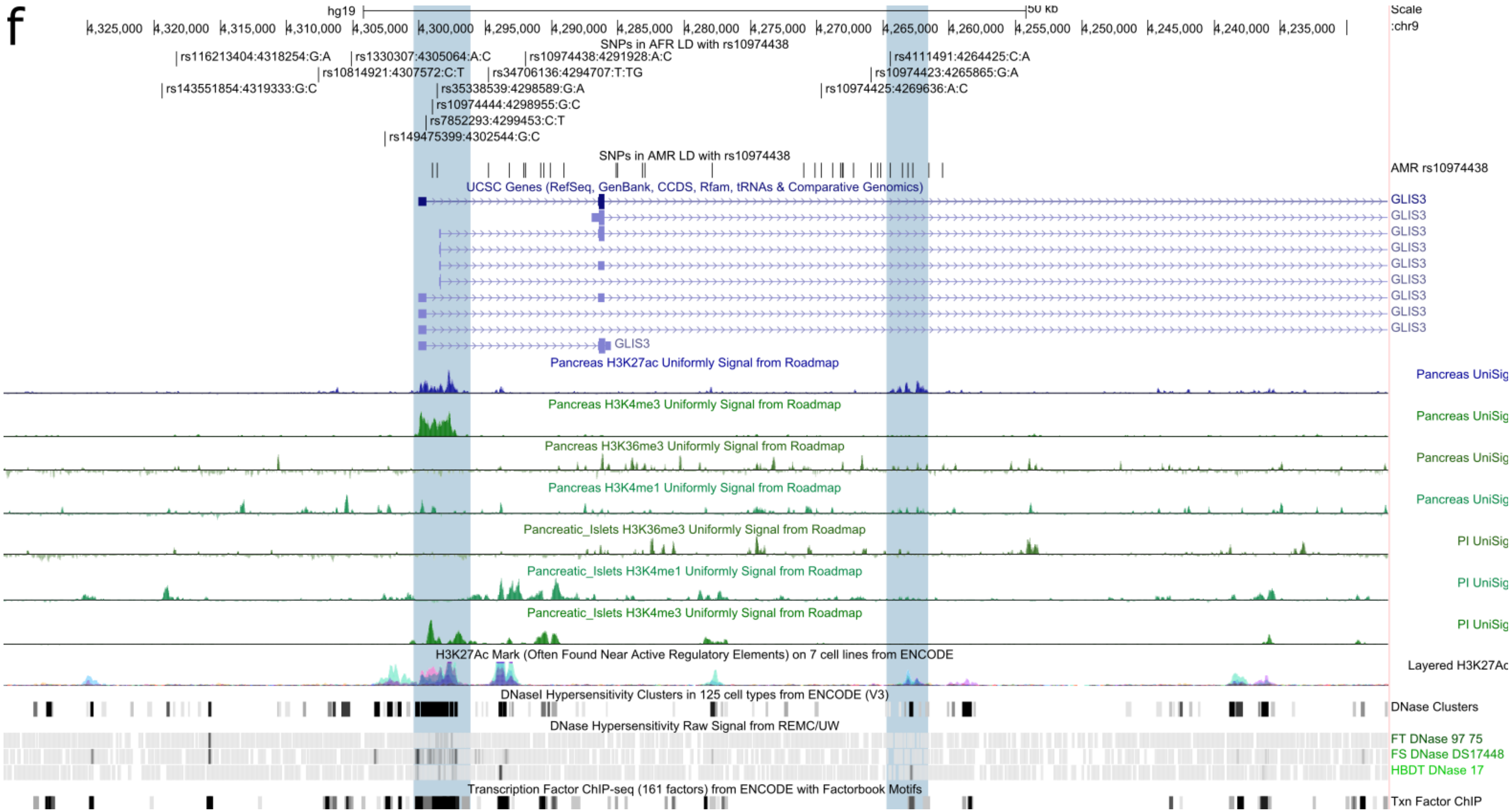

hg19
50 kb
Scale
chr9:
4,325,000
4,320,000
4,315,000
4,310,000
4,305,000
4,300,000
4,295,000
4,290,000
4,285,000
4,280,000
4,275,000
4,270,000
4,265,000
4,260,000
4,255,000
4,250,000
4,245,000
4,240,000
4,235,000
SNPs in AFR LD with rs10974438
rs116213404:4318254:G:A
rs1330307:4305064:A:C
rs10974438:4291928:A:C
rs4111491:4264425:C:A
rs10814921:4307572:C:T
rs34706136:4294707:T:TG
rs10974423:4265865:G:A
rs143551854:4319333:G:C
rs35338539:4298589:G:A
rs10974425:4269636:A:C
rs10974444:4298955:G:C
rs7852293:4299453:C:T
rs149475399:4302544:G:C
SNPs in AMR LD with rs10974438
AMR rs10974438
UCSC Genes (RefSeq, GenBank, CCDS, Rfam, tRNAs & Comparative Genomics)
GLIS3
GLIS3
GLIS3
GLIS3
GLIS3
GLIS3
GLIS3
GLIS3
GLIS3
GLIS3
Pancreas H3K27ac Uniformly Signal from Roadmap
Pancreas UniSig
Pancreas H3K4me3 Uniformly Signal from Roadmap
Pancreas UniSig
Pancreas H3K36me3 Uniformly Signal from Roadmap
Pancreas UniSig
Pancreas H3K4me1 Uniformly Signal from Roadmap
Pancreas UniSig
Pancreatic_Islets H3K36me3 Uniformly Signal from Roadmap
PI UniSig
Pancreatic_Islets H3K4me1 Uniformly Signal from Roadmap
PI UniSig
Pancreatic_Islets H3K4me3 Uniformly Signal from Roadmap
PI UniSig
H3K27Ac Mark (Often Found Near Active Regulatory Elements) on 7 cell lines from ENCODE
Layered H3K27Ac
DNaseI Hypersensitivity Clusters in 125 cell types from ENCODE (V3)
DNase Clusters
DNase Hypersensitivity Raw Signal from REMC/UW
FT DNase 97 75
FS DNase DS17448
HBDT DNase 17
Transcription Factor ChIP-seq (161 factors) from ENCODE with Factorbook Motifs
Txn Factor ChIP

g

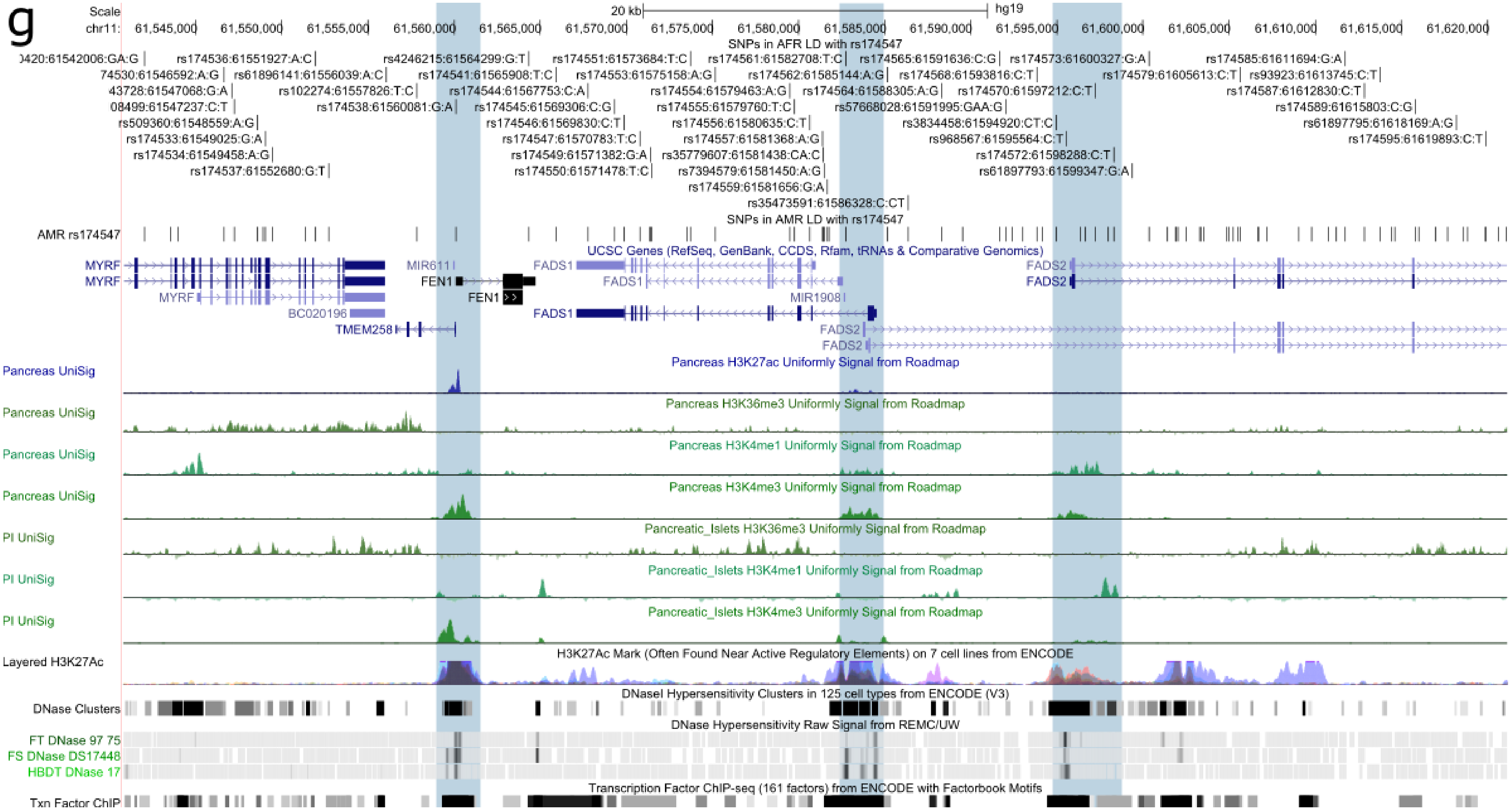
Scale
20 kb
hg19
chr11: 61,545,000 61,550,000 61,555,000 61,560,000 61,565,000 61,570,000 61,575,000 61,580,000 61,585,000 61,590,000 61,595,000 61,600,000 61,605,000 61,610,000 61,615,000 61,620,000
SNPs in AFR LD with rs174547
rs174536:61551927:A:C
rs4246215:61564299:G:T
rs174551:61573684:T:C
rs174561:61582708:T:C
rs174565:61591636:C:G
rs174573:61600327:G:A
rs174585:61611694:G:A
rs61896141:61556039:A:C
rs174541:61565908:T:C
rs174553:61575158:A:G
rs174562:61585144:A:G
rs174568:61593816:C:T
rs174579:61605613:C:T
rs93923:61613745:C:T
43728:61547068:G:A
rs102274:61557826:T:C
rs174544:61567753:C:A
rs174554:61579463:A:G
rs174564:61588305:A:G
rs174570:61597212:C:T
rs174587:61612830:C:T
08499:61547237:C:T
rs174538:61560081:G:A
rs174545:61569306:C:G
rs174555:61579760:T:C
rs57668028:61591995:GAA:G
rs174589:61615803:C:G
rs509360:61548559:A:G
rs174546:61569830:C:T
rs174556:61580635:C:T
rs3834458:61594920:CT:C
rs61897795:61618169:A:G
rs174533:61549025:G:A
rs174547:61570783:T:C
rs174557:61581368:A:G
rs968567:61595564:C:T
rs174595:61619893:C:T
rs174534:61549458:A:G
rs174549:61571382:G:A
rs35779607:61581438:CA:C
rs174572:61598288:C:T
rs174537:61552680:G:T
rs174550:61571478:T:C
rs7394579:61581450:A:G
rs61897793:61599347:G:A
rs174559:61581656:G:A
rs35473591:61586328:C:CT
SNPs in AMR LD with rs174547
AMR rs174547
UCSC Genes (RefSeq, GenBank, CCDS, Rfam, tRNAs & Comparative Genomics)
MYRF
MYRF
MYRF
BC020196
TMEM258
MIR611
FEN1
FEN1
FADS1
FADS1
FADS1
MIR1908
FADS2
FADS2
FADS2
FADS2
Pancreas UniSig
Pancreas H3K27ac Uniformly Signal from Roadmap
Pancreas UniSig
Pancreas H3K36me3 Uniformly Signal from Roadmap
Pancreas UniSig
Pancreas H3K4me1 Uniformly Signal from Roadmap
Pancreas UniSig
Pancreas H3K4me3 Uniformly Signal from Roadmap
PI UniSig
Pancreatic_Islets H3K36me3 Uniformly Signal from Roadmap
PI UniSig
Pancreatic_Islets H3K4me1 Uniformly Signal from Roadmap
PI UniSig
Pancreatic_Islets H3K4me3 Uniformly Signal from Roadmap
Layered H3K27Ac
H3K27Ac Mark (Often Found Near Active Regulatory Elements) on 7 cell lines from ENCODE
DNase Clusters
DNaseI Hypersensitivity Clusters in 125 cell types from ENCODE (V3)
DNase Hypersensitivity Raw Signal from REMC/UW
FT DNase 97 75
FS DNase DS17448
HBDT DNase 17
Transcription Factor ChIP-seq (161 factors) from ENCODE with Factorbook Motifs
Txn Factor ChIP

h

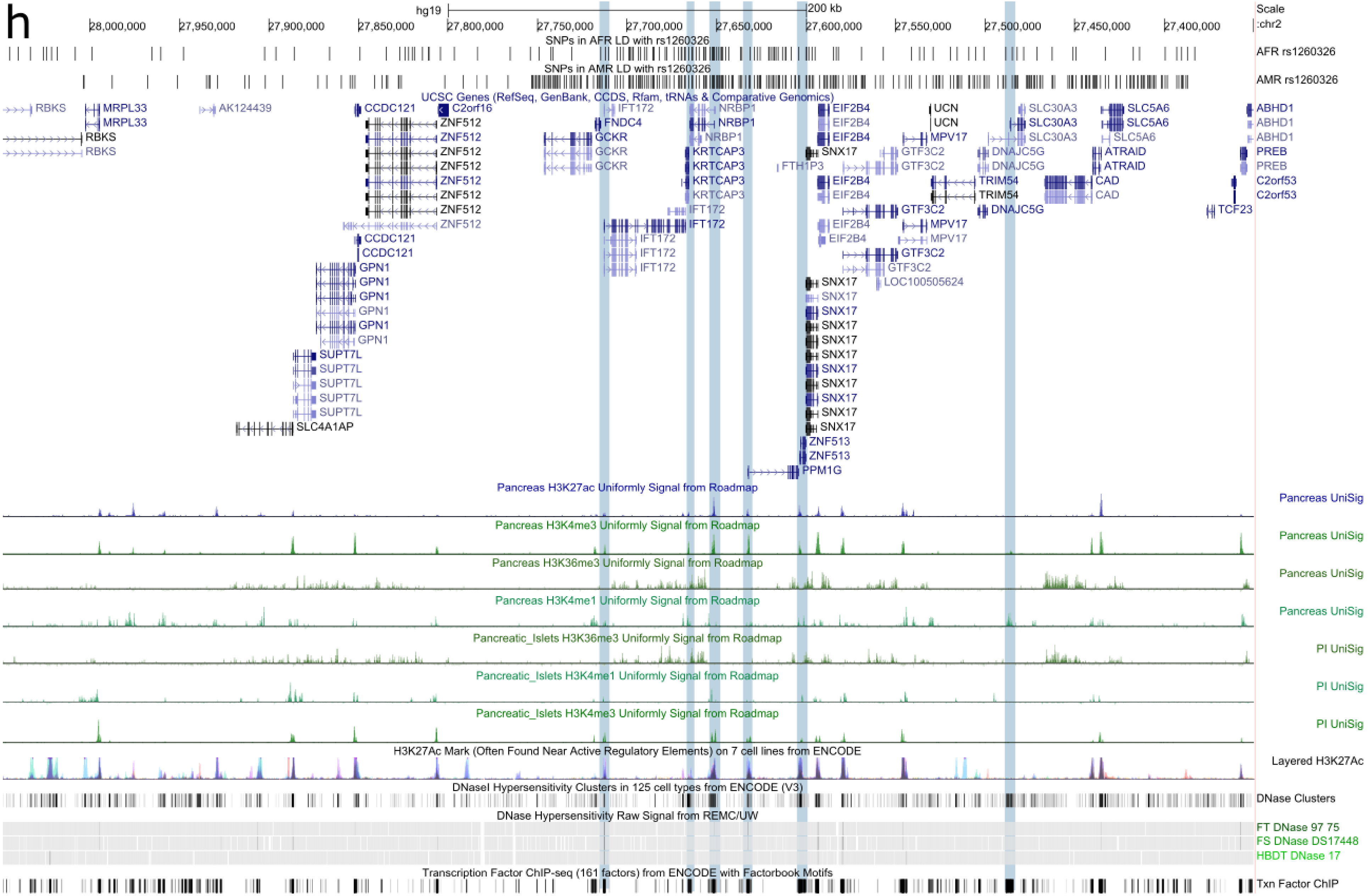

hg19
200 kb
Scale
chr2
28,000,000
27,950,000
27,900,000
27,850,000
27,800,000
27,750,000
27,700,000
27,650,000
27,600,000
27,550,000
27,500,000
27,450,000
27,400,000
SNPs in AFR LD with rs1260326
AFR rs1260326
SNPs in AMR LD with rs1260326
AMR rs1260326
UCSC Genes (RefSeq, GenBank, CCDS, Rfam, tRNAs & Comparative Genomics)
RBKS
MRPL33
AK124439
CCDC121
C2orf16
IFT172
NRBP1
EIF2B4
UCN
SLC30A3
SLC5A6
ABHD1
ZNF512
FNDC4
GCKR
KRTCAP3
FTH1P3
SNX17
GTF3C2
MPV17
DNAJC5G
ATRAID
PREB
TRIM54
CAD
C2orf53
TCF23
GPN1
SUPT7L
SLC4A1AP
LOC100505624
ZNF513
PPM1G
Pancreas H3K27ac Uniformly Signal from Roadmap
Pancreas UniSig
Pancreas H3K4me3 Uniformly Signal from Roadmap
Pancreas H3K36me3 Uniformly Signal from Roadmap
Pancreas H3K4me1 Uniformly Signal from Roadmap
Pancreatic_Islets H3K36me3 Uniformly Signal from Roadmap
PI UniSig
Pancreatic_Islets H3K4me1 Uniformly Signal from Roadmap
Pancreatic_Islets H3K4me3 Uniformly Signal from Roadmap
H3K27Ac Mark (Often Found Near Active Regulatory Elements) on 7 cell lines from ENCODE
Layered H3K27Ac
DNaseI Hypersensitivity Clusters in 125 cell types from ENCODE (V3)
DNase Clusters
DNase Hypersensitivity Raw Signal from REMC/UW
FT DNase 97 75
FS DNase DS17448
HBDT DNase 17
Transcription Factor ChIP-seq (161 factors) from ENCODE with Factorbook Motifs
Txn Factor ChIP

i

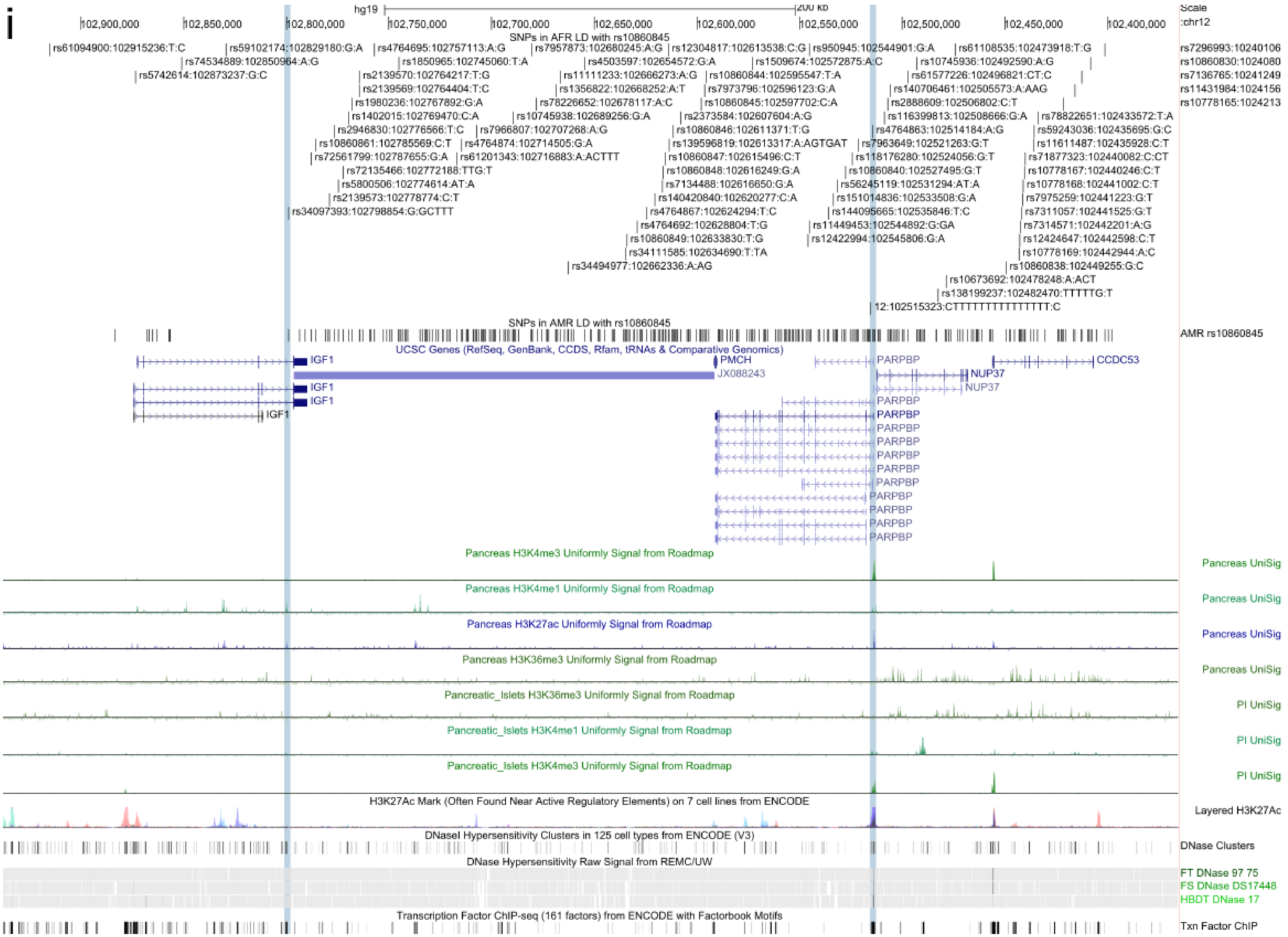

hg19
200 kb
Scale
chr12
102,900,000
102,850,000
102,800,000
102,750,000
102,700,000
102,650,000
102,600,000
102,550,000
102,500,000
102,450,000
102,400,000
SNPs in AFR LD with rs10860845
rs61094900:102915236:T:C
rs59102174:102829180:G:A
rs4764695:102757113:A:G
rs7957873:102680245:A:G
rs12304817:102613538:C:G
rs950945:102544901:G:A
rs61108535:102473918:T:G
rs74534889:102850964:A:G
rs1850965:102745060:T:A
rs4503597:102654572:G:A
rs1509674:102572875:A:C
rs10745936:102492590:A:G
rs5742614:102873237:G:C
rs2139570:102764217:T:G
rs11111233:102666273:A:G
rs10860844:102595547:T:A
rs61577226:102496821:CT:C
rs2139569:102764404:T:C
rs1356822:102668252:A:T
rs7973796:102596123:G:A
rs140706461:102505573:A:AAG
rs1980236:102767892:G:A
rs78226652:102678117:A:C
rs10860845:102597702:C:A
rs2888609:102506802:C:T
rs1402015:102769470:C:A
rs10745938:102689256:G:A
rs2373584:102607604:A:G
rs116399813:102508666:G:A
rs78822651:102433572:T:A
rs2946830:102776566:T:C
rs7966807:102707268:A:G
rs10860846:102611371:T:G
rs4764863:102514184:A:G
rs59243036:102435695:G:C
rs10860861:102785569:C:T
rs4764874:102714505:G:A
rs139596819:102613317:A:AGTGAT
rs7963649:102521263:G:T
rs11611487:102435928:C:T
rs72561799:102787655:G:A
rs61201343:102716883:A:ACTTT
rs10860847:102615496:C:T
rs118176280:102524056:G:T
rs71877323:102440082:C:CT
rs72135466:102772188:TTG:T
rs10860848:102616249:G:A
rs10860840:102527495:G:T
rs10778167:102440246:C:T
rs5800506:102774614:AT:A
rs7134488:102616650:G:A
rs56245119:102531294:AT:A
rs10778168:102441002:C:T
rs2139573:102778774:C:T
rs140420840:102620277:C:A
rs151014836:102533508:G:A
rs7975259:102441223:G:T
rs34097393:102798854:G:GCTTT
rs4764867:102624294:T:C
rs144095665:102535846:T:C
rs7311057:102441525:G:T
rs4764692:102628804:T:G
rs11449453:102544892:G:GA
rs7314571:102442201:A:G
rs10860849:102633830:T:G
rs12422994:102545806:G:A
rs12424647:102442598:C:T
rs34111585:102634690:T:TA
rs10778169:102442944:A:C
rs34494977:102662336:A:AG
rs10860838:102449255:G:C
rs10673692:102478248:A:ACT
rs138199237:102482470:TTTTTG:T
12:102515323:CTTTTTTTTTTTTTTT:C
rs7296993:10240106
rs10860830:1024080
rs7136765:10241249
rs11431984:1024156
rs10778165:1024213
SNPs in AMR LD with rs10860845
AMR rs10860845
UCSC Genes (RefSeq, GenBank, CCDS, Rfam, tRNAs & Comparative Genomics)
IGF1
PMCH
JX088243
PARPBP
CCDC53
NUP37
Pancreas H3K4me3 Uniformly Signal from Roadmap
Pancreas H3K4me1 Uniformly Signal from Roadmap
Pancreas H3K27ac Uniformly Signal from Roadmap
Pancreas H3K36me3 Uniformly Signal from Roadmap
Pancreatic_Islets H3K36me3 Uniformly Signal from Roadmap
Pancreatic_Islets H3K4me1 Uniformly Signal from Roadmap
Pancreatic_Islets H3K4me3 Uniformly Signal from Roadmap
Pancreas UniSig
PI UniSig
H3K27Ac Mark (Often Found Near Active Regulatory Elements) on 7 cell lines from ENCODE
Layered H3K27Ac
DNaseI Hypersensitivity Clusters in 125 cell types from ENCODE (V3)
DNase Clusters
DNase Hypersensitivity Raw Signal from REMC/UW
FT DNase 97 75
FS DNase DS17448
HBDT DNase 17
Transcription Factor ChIP-seq (161 factors) from ENCODE with Factorbook Motifs
Txn Factor ChIP

j

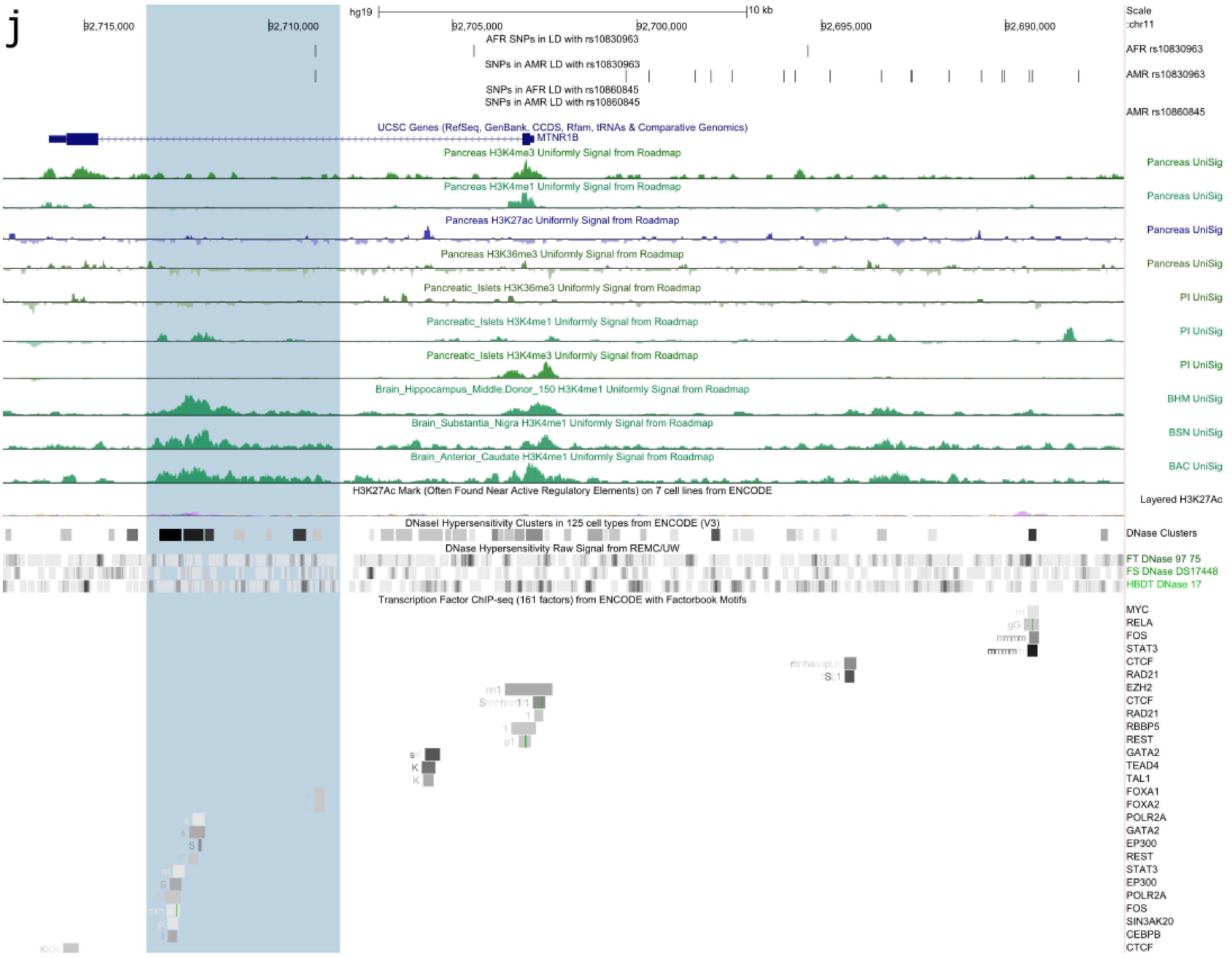
hg19
10 kb
Scale
chr11
92,715,000
92,710,000
92,705,000
92,700,000
92,695,000
92,690,000
AFR SNPs in LD with rs10830963
SNPs in AMR LD with rs10830963
SNPs in AFR LD with rs10860845
SNPs in AMR LD with rs10860845
AFR rs10830963
AMR rs10830963
AMR rs10860845
UCSC Genes (RefSeq, GenBank, CCDS, Rfam, tRNAs & Comparative Genomics)
MTNR1B
Pancreas H3K4me3 Uniformly Signal from Roadmap
Pancreas H3K4me1 Uniformly Signal from Roadmap
Pancreas H3K27ac Uniformly Signal from Roadmap
Pancreas H3K36me3 Uniformly Signal from Roadmap
Pancreatic_Islets H3K36me3 Uniformly Signal from Roadmap
Pancreatic_Islets H3K4me1 Uniformly Signal from Roadmap
Pancreatic_Islets H3K4me3 Uniformly Signal from Roadmap
Brain_Hippocampus_Middle.Donor_150 H3K4me1 Uniformly Signal from Roadmap
Brain_Substantia_Nigra H3K4me1 Uniformly Signal from Roadmap
Brain_Anterior_Caudate H3K4me1 Uniformly Signal from Roadmap
H3K27Ac Mark (Often Found Near Active Regulatory Elements) on 7 cell lines from ENCODE
DNaseI Hypersensitivity Clusters in 125 cell types from ENCODE (V3)
DNase Hypersensitivity Raw Signal from REMC/UW
Transcription Factor ChIP-seq (161 factors) from ENCODE with Factorbook Motifs
Pancreas UniSig
Pancreas UniSig
Pancreas UniSig
Pancreas UniSig
PI UniSig
PI UniSig
PI UniSig
BHM UniSig
BSN UniSig
BAC UniSig
Layered H3K27Ac
DNase Clusters
FT DNase 97 75
FS DNase DS17448
HBDT DNase 17
MYC
RELA
FOS
STAT3
CTCF
RAD21
EZH2
CTCF
RAD21
RBBP5
REST
GATA2
TEAD4
TAL1
FOXA1
FOXA2
POLR2A
GATA2
EP300
REST
STAT3
EP300
POLR2A
FOS
SIN3AK20
CEBPB
CTCF

k

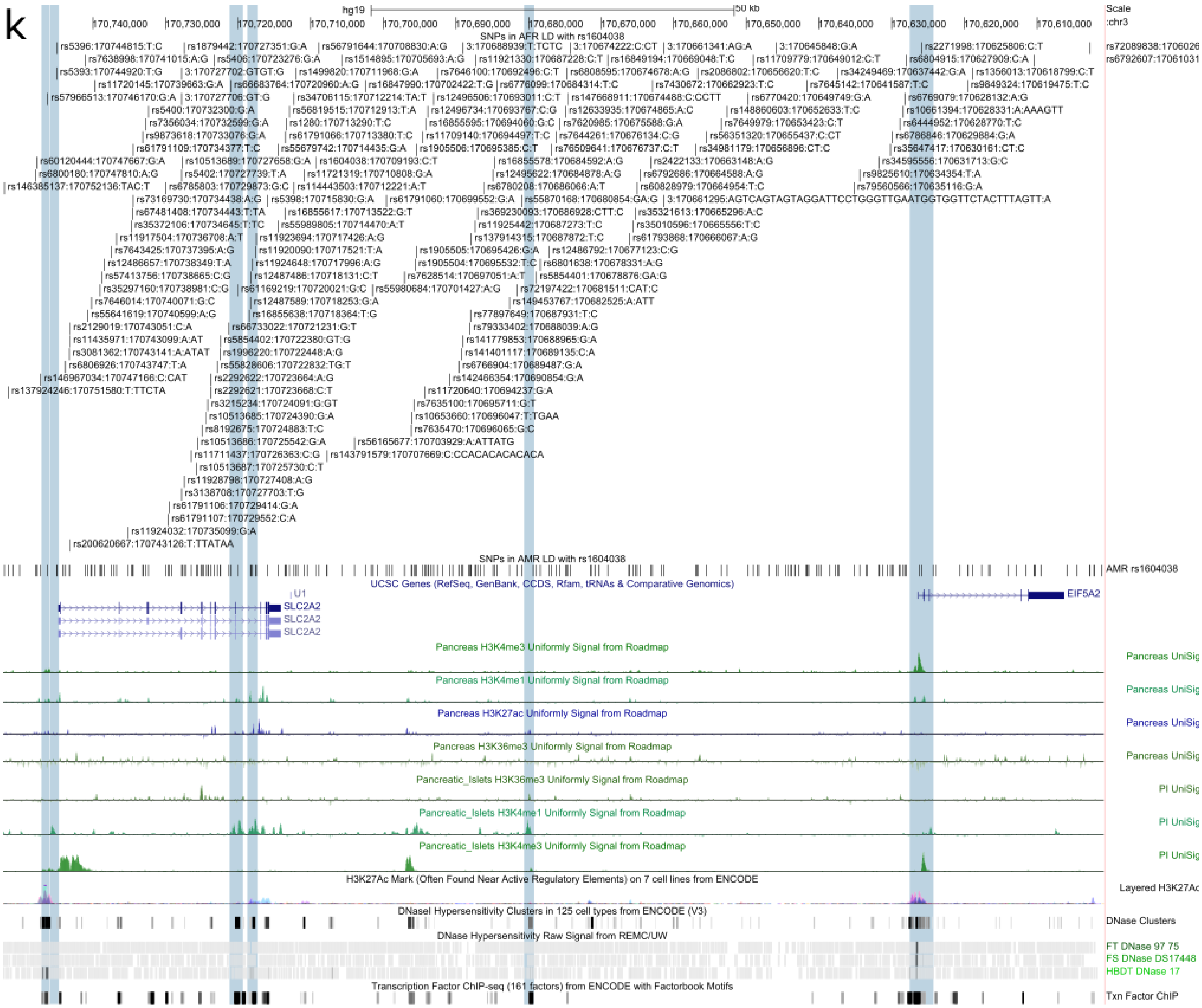

Scale
chr3
hg19
50 kb
170,740,000
170,730,000
170,720,000
170,710,000
170,700,000
170,690,000
170,680,000
170,670,000
170,660,000
170,650,000
170,640,000
170,630,000
170,620,000
170,610,000
SNPs in AFR LD with rs1604038
SNPs in AMR LD with rs1604038
AMR rs1604038
UCSC Genes (RefSeq, GenBank, CCDS, Rfam, tRNAs & Comparative Genomics)
U1
SLC2A2
SLC2A2
SLC2A2
EIF5A2
Pancreas H3K4me3 Uniformly Signal from Roadmap
Pancreas UniSig
Pancreas H3K4me1 Uniformly Signal from Roadmap
Pancreas UniSig
Pancreas H3K27ac Uniformly Signal from Roadmap
Pancreas UniSig
Pancreas H3K36me3 Uniformly Signal from Roadmap
Pancreas UniSig
Pancreatic_Islets H3K36me3 Uniformly Signal from Roadmap
PI UniSig
Pancreatic_Islets H3K4me1 Uniformly Signal from Roadmap
PI UniSig
Pancreatic_Islets H3K4me3 Uniformly Signal from Roadmap
PI UniSig
H3K27Ac Mark (Often Found Near Active Regulatory Elements) on 7 cell lines from ENCODE
Layered H3K27Ac
DNaseI Hypersensitivity Clusters in 125 cell types from ENCODE (V3)
DNase Clusters
DNase Hypersensitivity Raw Signal from REMC/UW
FT DNase 97 75
FS DNase DS17448
HBDT DNase 17
Transcription Factor ChIP-seq (161 factors) from ENCODE with Factorbook Motifs
Txn Factor ChIP

UCSC Genome Browser screen shots (http://genome.ucsc.edu, 24 August 2016) of the bioinformatic follow-up using Roadmap and ENCODE data are shown for eleven loci (a) *PROX1*, (b) *G6PC2*, (c) *ADCY5*, (d) *DGKB*, (e) *GCK*, (f) *GLIS3*, (g) *FADS2*, (h) G*CKR*, (i) *IGF1*, (j) *MTNR1B*, (k) *SLC2A2.* Methylation and acetylation modifications of the histone in metabolically relevant tissues (e.g. pancreatic islets) are shown. Regulatory elements within histone modifications are further demarked by open chromatin structure (DNaseI-seq), which is correlated with transcription factor binding. Finally, available ChIP-seq transcription factor binding sites are shown. Blue highlighting indicates a putative function element harboring either the lead SNP or SNPs in LD with the lead. Further details about eQTL, and specific transcription factors is given in ESM Table 7. In the *PROX1* locus (a), rs10494973, overlaps an intronic pancreatic enhancer and a *FOXA2* binding site, which is an essential activator of genes governing insulin secretion. In the *G6PC2* locus (b), rs560887 is a known functional splicing variant and bioinformatic annotation supports this variant as the strongest functional candidate. 9 LD SNPs in the *ADCY5* locus (c) overlap 4 pancreatic enhancers and were shown to be associated with *ADCY5* and *SEC22A* expression in multiple tissues. In the *DGKB* locus (d), 7 SNPs in LD with the lead, rs62448618, overlap a strong intergenic pancreatic islet enhancer. In the 7p13 locus (e), 7 variants in LD with the lead are positioned in 4 pancreatic islet enhancer/promoter regions that were also an eQTL for *GCK* in thyroid tissue. In the *GLIS3* locus (f), the lead SNP, rs10974438, is associated with expression of *GLIS3* in brain tissue and is in LD with 3 SNPs positioned in two pancreatic islet enhancers. In the *FADS3*-11q12.2 region (g), the lead SNP, rs174547, was in LD with 6 variants spanning 3 enhancer regions. In the *GCKR* region (h), the lead SNP, rs1260326, is a missense variant in exon 15 of *GCKR* that is predicted by SPANR to disrupt splicing and is associated with expression of *GCKR* in brain tissue. Additionally, 13 variants in LD with the lead span across six predicted enhancers. In the *IGF1* locus (i), rs10860845 tagged two putative functional variants positioned within two different pancreatic islet intronic enhancers. The lead SNP in the *MTNR1B* locus (j), rs10830963, falls in a brain and pancreatic islet/pancreas enhancers in the intronic region of *MTNR1B*, which also bind the transcription factors FOXA1 and FOXA2. In the *SLC2A2* locus (k), SNPs in LD with the lead SNP, rs1604038 are associated with *SLC2A2* and *EIF5A2* expression in brain tissue, overlap 7 pancreatic islet/pancreas enhancers, and one LD SNP was a missense variant predicted by SPANR to misregulate splicing of *SLC2A2*.

ESM Figure 4

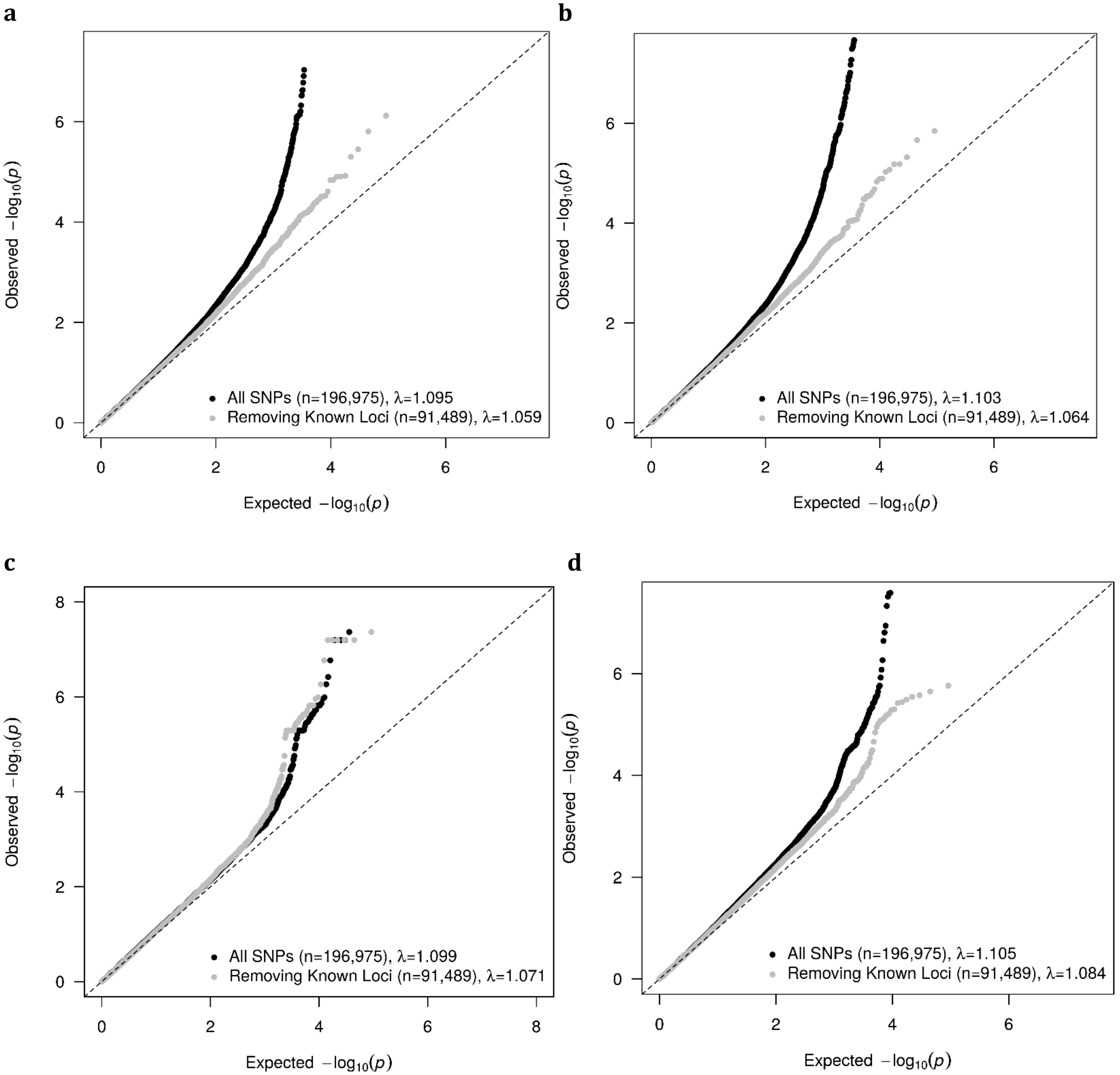

Quantile-Quantile (Q-Q) plots for Metabochip-wide fasting glucose and fasting insulin results. Expected and observed distributions of the –log10 of *p* values for Metabochip SNP associations with: fasting glucose before (a) and after (b) BMI adjustment, and natural log transformed fasting insulin before (c) and after (d) BMI adjustment. Gray colouring of SNPs show results after removing loci known to be associated with type 2 diabetes and associated biomarkers (fasting glucose, fasting insulin, HbA1C, 2 hour glucose), anthropometric traits like BMI, hip/waist ratio, and waist circumference, as well as levels of high/low density lipoprotein and triglycerides.

ESM Figure 5

a

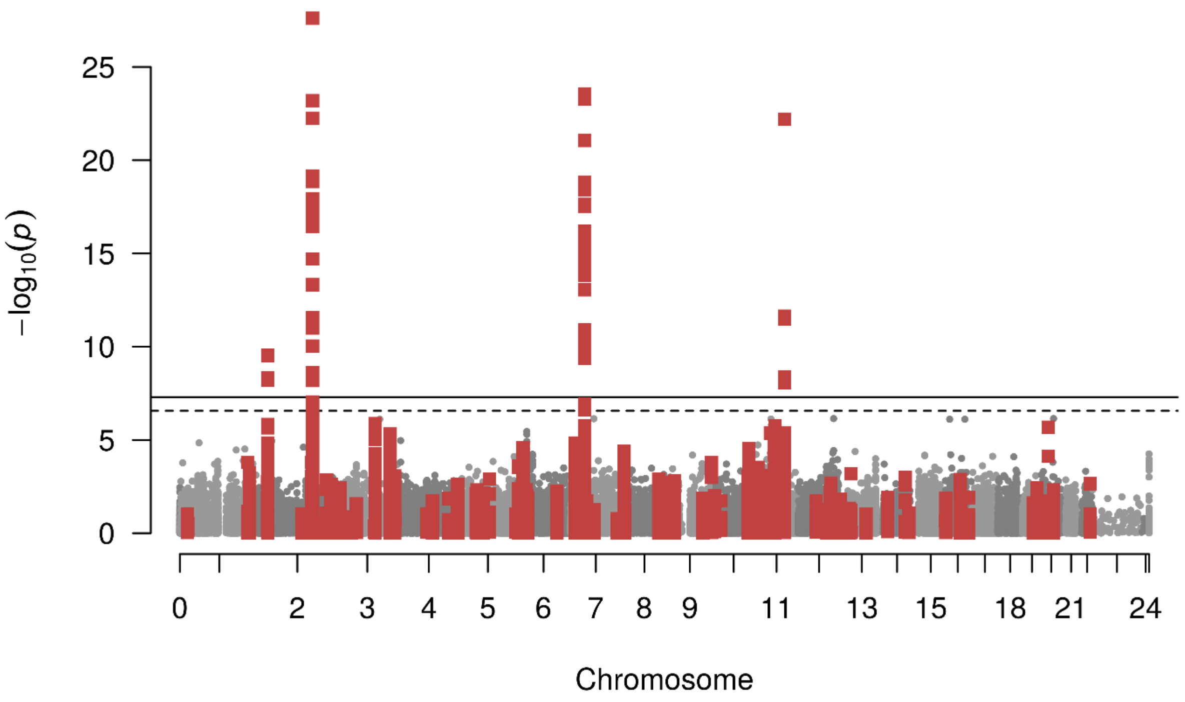

b

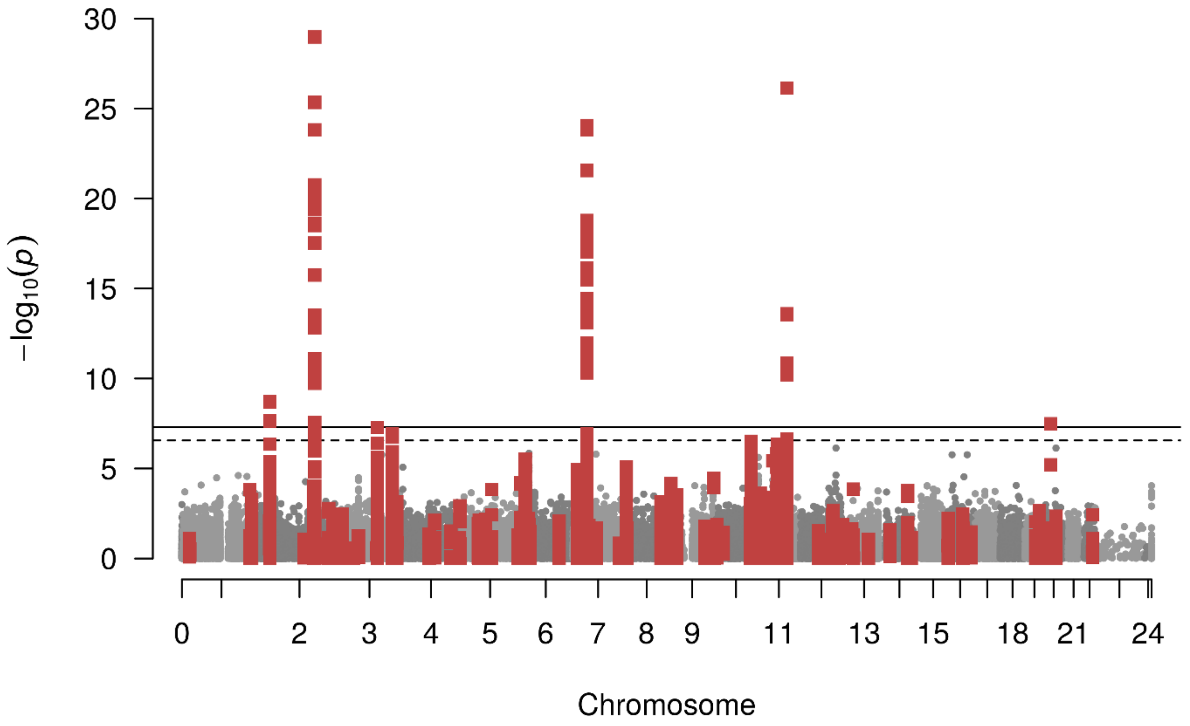

c

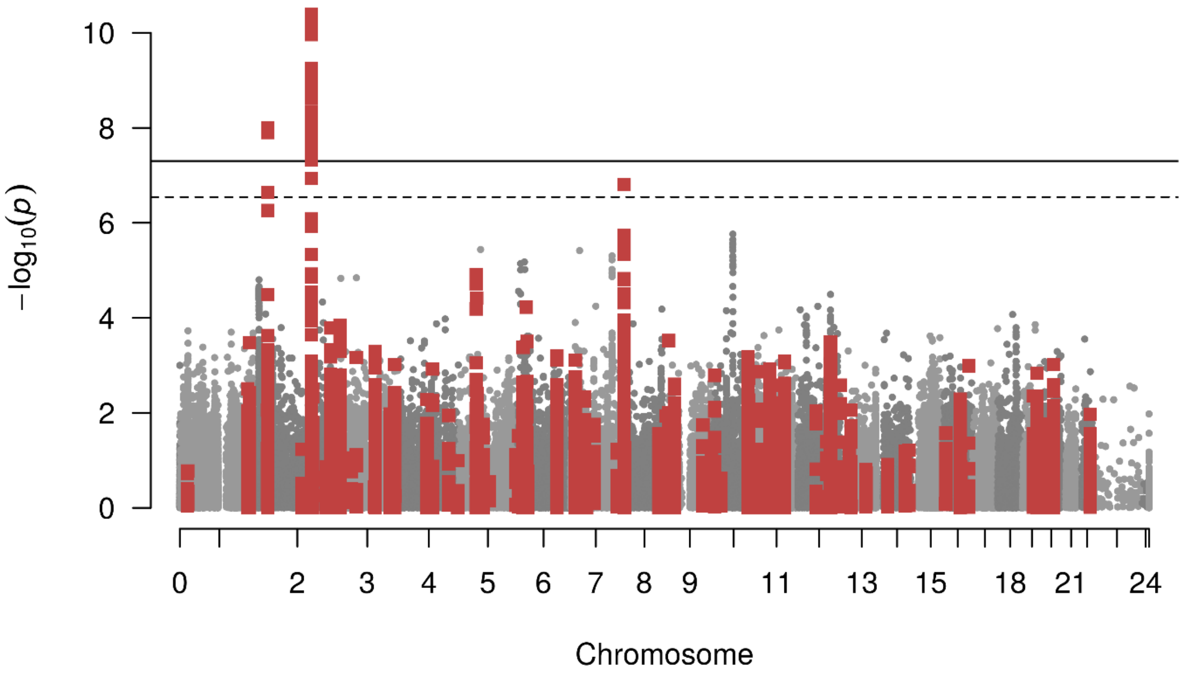

Fasting glucose and fasting insulin association *p* values for each Metabochip variant from the transethnic meta-analysis in models with and without BMI. The –log10 of *p* values for each SNP on the Metabochip is plotted against chromosomal positions. Fasting glucose model results with and without BMI covariate are shown in a) and b), respectively. Fasting insulin model results with BMI covariate are shown in c. Gray

and dark gray colouring of SNPs alternates by chromosome. Dark blue dots represent the SNPs in previously reported glycaemic trait loci (within 1 Mb of index SNP). Green dots correspond to novel (unobserved for these models). The blue solid line is the threshold for Metabochip-wide significance $\alpha = 2.5 \times 10^{-7}$; dashed blue line is threshold for genome-wide significance $\alpha = 5.0 \times 10^{-8}$.
